# The experiences of spirituality among adults with mental health difficulties: a qualitative systematic review

**DOI:** 10.1017/S2045796019000234

**Published:** 2019-05-03

**Authors:** K. Milner, P. Crawford, A. Edgley, L. Hare-Duke, M. Slade

**Affiliations:** University of Nottingham, Nottingham, UK

**Keywords:** Mental health, mental illness stigma, psychiatric services, rehabilitation, religion

## Abstract

**Aims:**

Despite an increasing awareness of the importance of spirituality in mental health contexts, a ‘religiosity gap’ exists in the difference in the value placed on spirituality and religion by professionals compared with service users. This may be due to a lack of understanding about the complex ways people connect with spirituality within contemporary society and mental health contexts, and can result in people's spiritual needs being neglected, dismissed or pathologised within clinical practice. The aim of this qualitative systematic review is to characterise the experiences of spirituality among adults with mental health difficulties in published qualitative research.

**Methods:**

An electronic search of seven databases was conducted along with forward and backward citation searching, expert consultation and hand-searching of journals. Thirty-eight studies were included from 4944 reviewed papers. The review protocol was pre-registered (PROSPERO:CRD42017080566).

**Results:**

A thematic synthesis identified six key themes: Meaning-making (sub-themes: Multiple explanations; Developmental journey; Destiny *v*. autonomy), Identity, Service-provision, Talk about it, Interaction with symptoms (sub-themes: Interactive meaning-making; Spiritual disruption) and Coping (sub-themes: Spiritual practices; Spiritual relationship; Spiritual struggles; Preventing suicide), giving the acronym MISTIC.

**Conclusions:**

This qualitative systematic review provides evidence of the significant role spirituality plays in the lives of many people who experience mental health difficulties. It indicates the importance of mental health professionals being aware of and prepared to support the spiritual dimension of people using services. The production of a theory-based framework can inform efforts by health providers to understand and address people's spiritual needs as part of an integrated holistic approach towards care.

## Introduction

Spirituality and religion are fundamental to many people's lives, health and wellbeing and are crucial for the effective delivery of holistic and person-centred care because they address issues of hope, meaning and purpose (Swinton, 2001). There has been a growing interest in spirituality within healthcare practice, research and policy, viewed now as an ethical obligation of professional care (Vermandere *et al*., 2011). Within psychiatry, attitudes have changed as the profession has become more accepting of the spiritual and religious concerns of patients (Sims and Cook, 2009). In 2015, the Executive Committee of the World Psychiatric Association accepted a position statement that the consideration of patients' spirituality, religious beliefs and practices and their relationship to the diagnosis and treatment of psychiatric disorders should be considered as essential components of psychiatric history taking, training and professional development (Moreira-Almeida *et al*., 2016; Verhagen, 2017).

Spirituality refers to the diverse and personal ways people seek meaning, purpose and connection in their lives (Gilbert, 2011*a*). Spirituality is often understood in a broader and more personally-defined way than religion, which is described as a system of faith or worship which seeks to understand the world and includes a transcendent being or beings and a meta-narrative (Gilbert, 2011*b*). There is however great scope and variability of both terms, for example, identifying spirituality and religion as synonymous or overlapping concepts, as contrasting or opposed, or defining spirituality as an over-arching term which includes both religious and non-religious expressions (Koenig *et al*., 2001; Royal College of Psychiatry, 2011). This review includes all variations of these terms.

In many societies, mental health problems are of increasing concern and can result in higher rates of discrimination, poverty, disease and mortality, leading to significant economic consequences (Kessler *et al*., 2009; WHO, 2013; Isaksson *et al*., 2018). An important development within mental health care policy and discourse internationally has been to focus attention on the stories and needs of people who experience mental health difficulties (Slade *et al*., 2012). This ‘recovery’ approach promotes person-centred and holistic interventions and understandings of mental health as well as the expertise of personal experience. Within this approach, biological and pharmacological frames of understanding are viewed alongside other aspects vital to recovery such as connection, hope, identity and meaning in life (Leamy *et al*., 2011).

Empirical research findings indicate a high prevalence of spirituality and religiosity among adults with severe mental illness (Bussema and Bussema, 2007; Russinova and Cash, 2007) and that religion and spirituality can have both positive and negative associations with health. Systematic reviews of the academic literature, which have identified more than 3000 empirical studies investigating the relationship between religion, spirituality and health, provide substantial evidence that the majority of studies exploring this relationship demonstrate that spiritual and religious beliefs and practices result in positive mental, physical and social health outcomes (Koenig *et al*., 2001; Koenig, *et al*., 2012). Research has also shown positive effects of spirituality and religion on various indicators of recovery from mental illness, for example, lower suicide rates (Jarbin and von Knorring, 2004) and lower levels of depressive symptoms (Bosworth *et al*., 2003).

The mechanisms by which spirituality and religion may facilitate mental health and recovery are varied and complex (Fallot, 2007; Webb *et al*., 2011). For example, spirituality may offer a way to cope with symptoms and difficulties (Corrigan *et al*., 2003; Pargament, 2011) by serving as a stress-buffering function (Webb *et al*., 2011), instilling a sense of hope (Bussema and Bussema, 2000) or offering a perspective of oneself outside the ‘sick role’ (Wilding *et al*., 2005). Religion and spirituality can also have challenging effects and associations such as spiritual struggles in which conflict can arise in relation to spiritual matters and which have been associated with poorer functioning (Exline, 2013). Additional negative effects include feelings of excessive guilt, abuse by religious advocates (Weaver and Koenig, 2006), rejection or stigma from religious communities (Fallot, 2007) and religious content becoming intertwined with psychiatric symptoms (Clarke, 2010). Lomax and Pargament (2016) argue that because of the double-sided capacity of spirituality to both foster and impede mental health and wellbeing, there is a need for more knowledge and understanding of this concept's multi-dimensional, multi-functional and dynamic character.

Being spiritual and being diagnosed with a mental illness can serve as a double stigma (Lukoff, 2007). Both service user narratives (e.g. Chadwick, 2007; Basset and Stickley, 2010) and research repeatedly show that many people would like their spirituality and religious needs addressed within healthcare contexts (e.g. Mental Health Foundation, 2002) yet find this aspect tends to be ignored, dismissed or pathologised by professionals (Zinnbauer and Pargament, 2000; Halasz, 2003; Cook, 2011). A ‘religiosity gap’ has been identified in empirical studies that show relatively lower levels of religious and spiritual beliefs among healthcare practitioners, and significant undervaluation of the importance of spiritual factors in recovery (Dein *et al*., 2010). Healthcare practitioners also report a lack of clear practical guidance about spirituality and barriers in knowledge, skills and confidence in addressing such needs (Fallott, 2007; Mooney, 2009). Within such contexts, spiritual needs may be frequently interpreted as mental illness, thereby shutting down further discussions about spiritual care (Greasley *et al*., 2001; Mental Health Foundation, 2002) and leading to a reluctance to engage with psychiatric services (Dein *et al*., 2010).

One of the difficulties clinicians may experience when working with service users' spiritual needs is that the relationship between spirituality and mental health problems can be complex. Research findings indicate the need for a more refined understanding of the interplay between spirituality and mental health and the careful exploration of this subject with people who experience mental health difficulties (Moreira-Almeida *et al*., 2014).

Systematic reviews of qualitative studies are an emerging methodology aimed at providing comprehensive understandings of social phenomena across a diverse range of contexts and are increasingly viewed as high-level evidence to underpin clinical practice guidelines (Tong *et al*., 2014). To date, there are no qualitative systematic reviews that explore the experiences of spirituality and mental health difficulties. This review aims to systematise the spirituality and mental health care literature by identifying and discussing emerging themes within qualitative research studies of the experiences of spirituality among people with mental health difficulties.

## Methods

### Design

A qualitative systematic review was undertaken. The review protocol was pre-registered (PROSPERO:CRD42017080566). Protocol deviations were: extending Medline to include exploded terms to increase sensitivity, reducing duplicates in search terms and extending the date range to capture recent research.

### Eligibility criteria

The inclusion criteria were: qualitative design; participants aged 18 years or over; current or previously diagnosed or self-reported mental health difficulties; self-defined spiritual or religious beliefs; peer-reviewed studies with the main focus on spirituality/religion; English language. To address the broad scope of this systematic review and a large number of studies found during an initial explorative search, we excluded those focusing on conditions, contexts, phenomena or groups of people which might produce results specific to those situations and warrant study in their own right. For a full list of exclusion criteria, see online Supplementary material 1.

### Search strategy and information sources

A systematic search of seven electronic databases was conducted from inception to 21 September 2018: MEDLINE, PsycINFO, AMED (all accessed via Ovid), ASSIA (Proquest), CINAHL and ATLA (both EBSCO) and Web of Science (1900 onwards). Further sources were forward and backward citation searching, expert consultation and hand-searching (as a supplement to online database searching) of the following journals congruent with the search focus from 2000 to February 2018: *Mental Health, Religion and Culture*; *Psychiatric Rehabilitation Journal*; *International Journal for the Psychology of Religion*; and *Journal for the Study of Spirituality*.

A full search strategy for MEDLINE (Ovid, In-Process and Other Non-Indexed Citations 1946 to Present) including search terms used is shown in online Supplementary material 2. Following the search, all identified citations were collated and uploaded into Endnote and duplicates removed. Titles and abstracts were screened against inclusion criteria, with 400 screened independently and any disagreements resolved through discussion. Full texts of selected citations were assessed against inclusion criteria, with 10% also screened by an independent reviewer.

### Data extraction and quality appraisal

The data extraction table is shown in online Supplementary material 3. The quality of studies was rated using the CASP checklist for qualitative research (Critical Appraisal Skills Programme, 2017). This is a clear and straightforward checklist comprising of ten questions relating to the design of the study. A second independent reviewer assessed a selection (10%) of the papers and any disagreements were resolved through discussion. As there is little evidence about decisions to exclude studies on the basis of their quality (Thomas and Harden, 2008) all studies were included.

### Data analysis and synthesis

Study findings were synthesised using a thematic synthesis approach based on Braun and Clarke's (2006) thematic analysis which involves six phases of generating codes and searching for and refining themes. The analysis was also informed by Thomas and Harden's (2008) thematic synthesis which is specifically designed for bringing together and integrating the findings within a qualitative systematic review. The final themes were reviewed by a second independent researcher and discrepancies were resolved through discussion. In addition, the analysis and theme framework were discussed with the co-authors who have expertise in a range of research and scholarly backgrounds. To address reflexivity and enhance the credibility and trustworthiness of the research, an audit trail and a reflexive journal were kept throughout the research process.

## Results

### Study selection

**Fig. 1.**
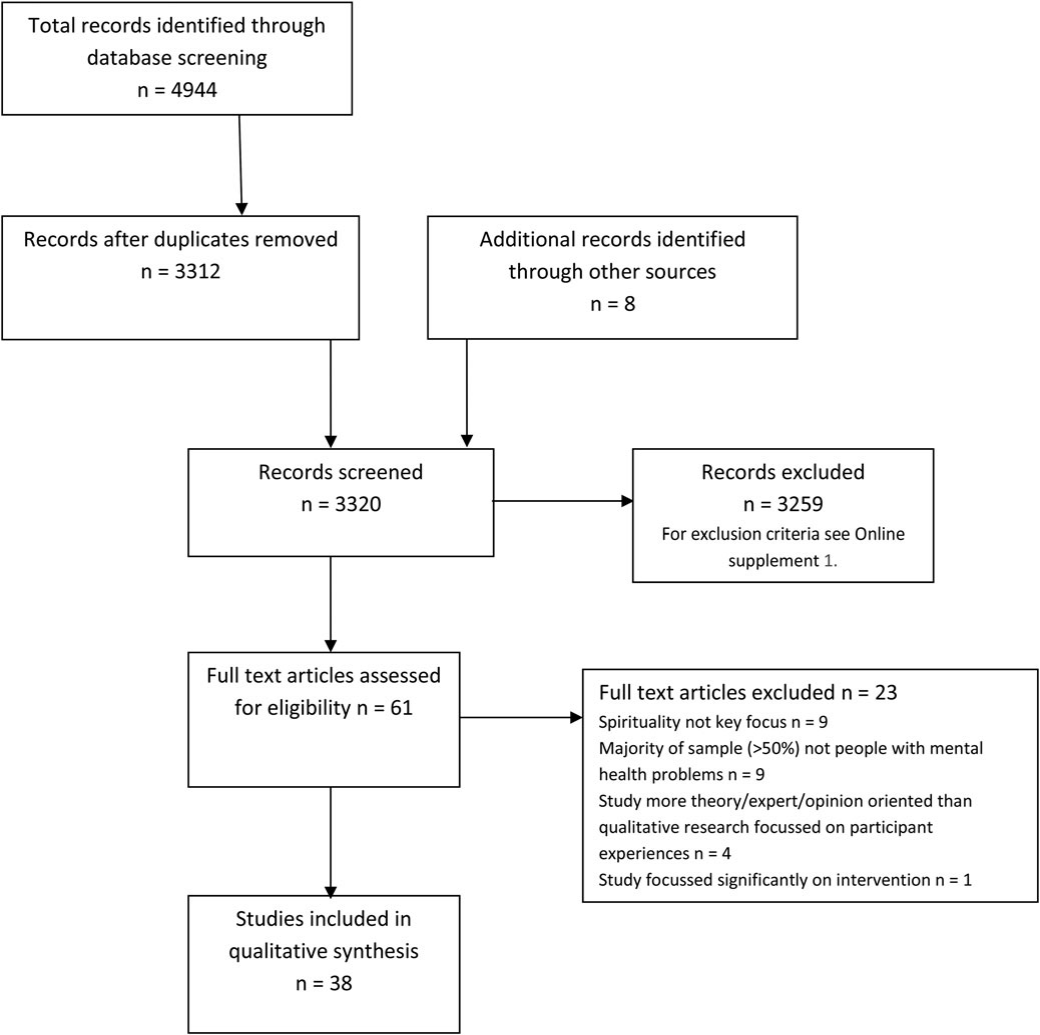
Flow diagram of study search and selection process.

Results of the search and screening processes are shown in Fig. 1.

The data extraction table for the 38 included studies is shown in online Supplementary material 3. Included studies involved a total sample of 594 participants and came from 15 countries: USA (*n*  =  10), UK (*n*  =  7), Canada (*n*  =  4), Australia (*n*  =  3), two from each of Norway, Sweden and the Netherlands and one from each of Switzerland, Ireland, Saudi Arabia, Jordan, Brazil, India, Taiwan and Malaysia.

A broad range of religious and spiritual beliefs and affiliations were investigated including major religions, particularly Christianity and Islam. Many participants did not identify with one specific religion and used more flexible classification systems. They had experienced a range of mental health problems and severity of symptoms.

Quality appraisals for the different studies are shown in the data extraction table in the online Supplementary material. Studies were rated against each of ten questions and then allocated to low (0–4), medium–low (5–5.5), medium (6–6.5), medium–high (7–7.5) and high (8–10) quality. The majority of studies had a rating of 7 and above (23 studies). Although non-standardised, this assessment identified the majority of studies as well designed. Higher quality studies had more comprehensive themes and contributed most to the synthesis.

### Results of thematic synthesis

Twelve initial themes were derived which were then reviewed against the data set, further refined and combined to form six overarching themes and nine sub-themes, described, along with examples, in Table 1.

**Table 1.** Theme descriptions and illustrative quotes

Theme and subtheme	Description	Illustrative quotes
**1. Meaning-making**	The ways in which people utilise their religious and spiritual beliefs to try to make sense of their experiences of mental illness.	‘…when you don't know, it's harder to deal with. When you know it's a lot easier to deal with.’ (Heffernan *et al*., 2016, p. 352)
1.1 Multiple-explanations	The often contradictory explanatory frameworks that people grapple with to try to make sense of their mental health difficulties – e.g. religious/spiritual *v*. medical/clinical, which may conflict and lack integration.	‘The most conflicting message I had was the diagnosis of the psychosis like people saying it's ok God's got it in hand, everything's gonna be fine, and then they're saying no you're psychotic you need medication, other people saying no you don't need medication, yes you do, no you don't…. will somebody please just tell me what is going on.’ (Heffernan *et al*., 2016, p. 352)
1.2 Developmental journey	The ways in which people's perceptions about and relationships with spirituality are dynamic over time, e.g. changes in the ways spirituality is experienced, valued and expressed.	‘I tend to err on the side of it being a transformational process. So that I can work with it. Because if it's a mental illness, it shuts me down.’ (Nixon *et al*., 2010, p. 538)
		‘One of the things that I have actually gained from having a mental illness (is) that I…have looked at what this God stuff means.’ (Wilding *et al*., 2006, p.147)
1.3 Destiny *v*. autonomy	The level of choice and control people conceptualise themselves as having in relation to their religious or spiritual belief systems. On one end of this spectrum is the sense that what is happening is divinely intentioned, or as involving a sense of fate or destiny. On the other hand, a sense of choice and control, agency and freedom are important for some.	‘The illness is Allah's will … It is a trial … I pray to Allah everyday to make me feel better.' (Eltaiba and Harries, 2015, p. 732)
		‘Ming (fate) arranges our lives. If I have to stay in the hospital and suffer from this illness, that is my Ming.’ (Yang *et al*., 2012, p. 361)
		‘…it should be up to that person to have that choice… if you have belief in yourself and believe what you believe then it's not put into you… it's not forced.’ (Heffernan *et al*., 2016, p. 350)
**2. Identity**	The centrality of spirituality for many people's lives and core sense of self. Spirituality represents for many the core essence of who they are, shaping their identities through their experiences of illness, struggle, recovery and meaning-making. Participants draw on their spiritual frameworks to develop and negotiate a spiritual identity.	‘…to invalidate a person's spirituality no matter how distorted that is, is to invalidate that real core sense of self and I think once you do that you risk doing untold damage to somebody.’(Mental Health Foundation, 2002, p. 22).
		‘I just want to be loved for who I am.’ (Mental Health Foundation, 2002, p. 46)
**3. Service provision**	The ways in which people's spiritual needs are addressed or not within mental health care services and how people describe their interactions with services and mental health professionals.	‘I felt very alone and isolated in a strange environment, one which I hadn't experienced before and things were happening to me that I didn't know… I wanted some kind of stability within that and that was why my faith and religion were coming in at that time … I wanted to identify with it as soon as possible.’ (Mental Health Foundation, 2002, p. 20)
		‘(spiritual care's) very very important for mental health; sometimes it's the only thing that seems, that can maybe get through to someone. It's a different level of understanding, that goes beyond words, that goes beyond, something you can touch, it goes beyond all that…’ (Raffay *et al*., 2016, p. 5).
**4. Talk about it**	What people say they particularly need and yet may lack opportunities to do in relation to their experiences of spirituality and mental health difficulties, and how practitioners can better support people's spiritual needs.	‘Many people don't realise how devastating it is to be told that that's not real, that that's fantasy … Because to the people it is real and it needs to be treated as if it is real instead of just discarded and pushed aside, because it is a very big part of people … It's their core’ (Starnino, 2014, p. 127).
		‘The community psychiatric nurse was terrific. Although he was not a Christian, he asked me very, very pertinent questions about how I could reconcile my faith with what was happening to me and what God meant to me.’ (Mental Health Foundation, 2002, p. 23)
**5. Interaction with symptoms**	This theme describes how people's symptoms or mental health difficulties interact with their spirituality, sometimes in quite challenging and disruptive ways.	
5.1 Interactive meaning-making	Often experienced as a clash of multiple or different realities which might involve experiencing visions, hearing voices from nonphysical entities or other unusual religious experiences or beliefs.	‘Even, to some extent, the spiritual stuff, as embarrassing as it is. I say embarrassing because I sort of, I don't really believe in a corporeal, nonbodied being [God]. I just don't believe that. On some level I don't believe that, and yet, I've experienced demon possession.’ (Jones *et al*., 2016, p. 494)
5.2 Spiritual disruption	The ways in which mental health symptoms sometimes intersect with people's spirituality resulting in challenging experiences or impeding people's ability to connect with their spirituality and spiritual practices.	‘The obsessions started last year, I started having thoughts about my faith, about the prophet, about God's creation. Then I started having doubts about making mistakes in my prayers or when I read verses of the Qura'an. I started repeating my prayers so many times that I had to miss school on many occasions.’ (Al-Solaim and Loewenthal, 2011, p. 175)
		‘…sometimes I lose my focus… I become quite lazy in sort of worshipping and doing my prayers and I can't concentrate.’ (Heffernan *et al*., 2016, p. 349)
**6. Coping**	This theme refers to the many and varied ways in which people utilise their spirituality to help them to deal with the challenges of their mental health problems. The process of coping may be experienced as an active process involving making persistent efforts to engage with spirituality and religious practices and to gain skills and acquire information. This process can be challenging and impeded by symptoms or struggles.	‘To improve things you need different skills. Skills could be acquired and could be lost with illness. I need to work on gaining skills and to change.' (Eltaiba and Harries, 2015, p. 731)
6.1 Spiritual practices	People engage with a variety of spiritual practices (e.g. prayer, meditation, mindfulness, attending a place of worship or quiet space, or reading religious or spiritual texts) to help them to cope with their mental health difficulties. These help people to strengthen their connection with their spiritual lives.	‘I feel really sad when bad things happen, but I sit, listen to the Qura'an, and feel better. My family is the same. Religion helps you adapt, prayer distances Satan from me and the more I pray the more God protects me.’ (Al-Solaim and Loewenthal, 2011, p. 178)
		‘Whenever I have a problem, I pray and fast.’ (Al-Solaim and Loewenthal, 2011, p. 179)
6.2 Spiritual relationship	People's relationship with God or a higher spiritual power is often central to their faith. It is described as the most important relationship of some people's lives and for this reason has crucial importance for coping during times of illness. This relationship can provide a sense of comfort, reassurance, protection, guidance and security as well as feelings of peace, strength, courage and the ability to feel more positive.	‘God became a friend… everything, I discussed with God… (He) put up with loads … it's how I survived.’ (Mental Health Foundation, 2002, p. 32)
		‘I really don't look to people. I look to God – because people are not able – they're able to help a certain amount, but the Lord has been my true strength … God has seen me through everything.’ (Sullivan, 1993, p. 130)
		‘It's God who helps me, I ask him for solutions and slowly he helps me, giving me the answers.’ (Salimena *et al*., 2016, p. 4)
6.3 Spiritual struggles	Sometimes people experience spiritual struggles or difficulty finding ways to cope. Common challenges include feelings of guilt or shame, or of stigma from spiritual communities.	‘I have actually said to God, why don't you just leave me … It was more comfortable when I didn't know.’ (Mental Health Foundation, 2002, p. 16)
		‘I have been told by a minister that he does not want the mentally ill in his church.’ (Moller, 1999, p. 8)
6.4 Preventing suicide	Sometimes people's faith, spirituality or relationship with God is the very thing that keeps them alive or from hurting themselves during their most difficult struggles with mental health problems.	‘God has saved my life.’ ‘Probably the biggest impact about my belief in God is when I have been suicidal… my faith has probably been the thing that's most kept me from hurting myself.’ (Rajakumar *et al*., 2008, p. 96)
		‘If I had no faith, I don't know how I'd get through it. No faith, no hope, no light at the end of the tunnel. I would end it.’ (Drinnan and Lavender, 2006, p. 324)

The six themes of *Meaning-making*, *Identity*, *Service-provision*, *Talk about it*, *Interaction with symptoms* and *Coping* form the acronym ‘MISTIC’ and are summarised briefly with their sub-themes below and in more detail in online Supplementary material 4.

### Description of themes

#### Meaning-making

The theme *Meaning-making* refers to the ways in which people utilised their spiritual beliefs to try to make sense of their experiences of mental illness. This was one of the most frequently occurring themes, evident in 33 studies, and had three sub-themes, *Multiple explanations*, *Developmental journey* and *Destiny v*. *autonomy*.

The sub-theme of *Multiple explanations* describes the often contradictory explanatory frameworks that people grappled with to try to make sense of their mental health experiences (Heffernan *et al*., 2016). Because of the lack of integration between conflicting views held by mental health services and religious organisations (Baker, 2010) participants sometimes struggled to arrive at an explanatory framework, which could in turn impede recovery. Within the sub-theme *Developmental journey*, some studies showed a change or maturation in the ways participants experienced, valued and expressed their spirituality over time (Wilding *et al*., 2006; Marsden *et al*., 2007; Starnino and Canda, 2014). Many participants viewed their spirituality as a journey involving phases of confusion and doubts, insights and opportunities for transformation (Mental Health Foundation, 2002). A final sub-theme *Destiny v. autonomy* concerned the level of choice and control people conceptualised themselves as having in relation to their religious or spiritual belief systems (Smith and Suto, 2012; Yang *et al*., 2012; Eltaiba and Harries, 2015).

#### Identity

*Identity* was a prominent theme (mentioned in 20 studies) and refers to the centrality of spirituality to many people's lives. Participants drew on their spiritual frameworks to develop and negotiate a spiritual identity (Drinnan and Lavender, 2006; Wilding *et al*., 2006). Spirituality was seen as vital to life and enabled many participants to develop a healthier more empowered view of themselves (Wilding *et al*., 2006; Starnino and Canda, 2014; Heffernan *et al*., 2016).

#### Service provision

The theme of *Service provision* was evident in 23 studies and relates to people's experiences of and interactions with mental health care services and professionals. The most commonly recurring experience under this theme was that participants felt their spiritual experiences were often dismissed, misunderstood or pathologised by professionals. Participants also expressed frustration at the lack of provision within services for their spiritual needs (Koslander and Arvidsson, 2007) and talked about ways mental health professionals and services could provide them, for example, by offering access to safe and quiet spaces where they could engage in spiritual practices (Mental Health Foundation, 2002).

#### Talk about it

The theme *Talk about it* was highlighted in 13 studies and related to what participants said they sometimes needed most in relation to their experiences of spirituality and mental health and what practitioners could helpfully do to support them but often did not.

One of the greatest challenges that participants struggled with during the meaning-making process was having to negotiate their experiences in relative social and cultural isolation (Jones *et al*., 2016). They wanted to talk to others to gain comfort and to understand the meaning of their ill-health in religious and spiritual terms (Mental Health Foundation, 2002; Macmin and Foskett, 2004). They looked to healthcare staff to help them with this because the complex interplay between their spirituality and mental health could be difficult to interpret alone and doing so could have adverse effects on recovery (Ouwehand *et al*., 2014; Heffernan *et al*., 2016). However, some participants feared that their spiritual experiences would be interpreted as symptoms of mental illness (Macmin and Foskett, 2004; Wilding *et al*., 2006). Participants found it helpful when practitioners listened, reassured and encouraged open discussions (Starnino, 2014).

#### Interaction with symptoms

*Interaction with symptoms* was a theme addressed by 18 studies. This theme describes how people's mental health difficulties or symptoms could interact with their spirituality, often in challenging or disruptive ways. Although a more complex theme, two distinctive sub-themes were identified. Firstly, *Interactive meaning-making* describes the ways in which the interaction between spirituality and mental health symptoms were connected with unusual experiences and the attempts to make meaning from these experiences. Secondly, *Spiritual disruption* describes how mental health symptoms could disrupt people's ability to engage in spirituality.

#### Coping

*Coping* was a prominent theme identified in 34 of the studies referring to the many ways people reported utilising their spirituality to deal with the challenges of their mental health problems. It has four sub-themes: *Spiritual practices*, *Spiritual relationship*, *Spiritual struggles* and *Preventing suicide*.

A variety of *Spiritual practices* helped participants to cope with their mental health problems with prayer having particular significance (Al-Solaim and Loewenthal, 2011; Eltaiba and Harries, 2015). People's *Spiritual relationship*, whether with God, a spiritual figure or a higher spiritual power was often significant for participants, being described as central to their faith or the most important relationship of their lives with crucial importance for coping during times of illness (Lilja, *et al*., 2016; Hanevik *et al*., 2017; Oxhandler *et al*., 2018). Heffernan *et al*. (2016) found that the role of a genuine reciprocal relationship with a spiritual figure was so essential that it influenced many other aspects of people's experiences and that recovery was impeded when the relationship was disrupted within hospital settings. Sometimes people experienced *Spiritual struggles* or difficulty finding ways to cope. Common challenges included feelings of guilt, shame or of stigma from spiritual communities. Perhaps the most striking sub-theme of coping, *Preventing suicide*, concerned how it was sometimes people's spirituality or religion which kept them alive during times of their most difficult struggles with mental health problems (Mental Health Foundation, 2002; Nixon *et al*., 2010; Hustoft *et al*., 2013).

## Discussion

This qualitative systematic review comprising a thematic synthesis of 38 studies from 15 countries and a range of belief systems and investigating experiences of spirituality in the context of mental health is the first of its kind. It presented six key themes characterising important experiences of spirituality among people with mental health difficulties. There was an amplificatory force to this review in its overlap with a previous systematic review identifying recovery processes comprising Connectedness, Hope, Identity, Meaning and Empowerment (the CHIME framework) (Leamy *et al*., 2011). Two CHIME processes map onto two key themes in this review: *Meaning-making* and *Identity.* This marks relatedness between the concepts of spirituality and recovery, which are both often defined in relation to finding meaning and purpose in life (Anthony, 1993; Gilbert, 2011*a*).

*Meaning-making* and *Coping* were prevalent themes in this study. There is a sizeable literature on religious coping (e.g. Pargament and Raiya, 2007; Lomax and Pargament, 2016) but less investigation around meaning-making from a psychiatric perspective despite the importance it has for people with mental health difficulties (Huguelet, 2017). Carl Jung was convinced that meaning, which he referred to as a ‘healing fiction’, had been underestimated in the approach to illness (Jung, 1956) and David Tacey (2013) argues that illuminating the processes of spiritual meaning-making may hold important keys to better understanding and assisting recovery of mental health difficulties. Existing spiritual development theories may illuminate the *Developmental journey* sub-theme of *Meaning-making*, such as faith development theory (Fowler, 1981) and the psycho-spiritual developmental framework (Culliford, 2014) which describes the process of spiritual growth as involving a renewed sense of meaning, often after encountering major adversity. Some authors argue that understanding and treating mental health difficulties as difficult stages in a natural development process can help facilitate recovery and spiritual development (Grof and Grof, 1989; Crowley, 2006; Clarke *et al*., 2016).

There is increasing evidence of the need for psychiatrists and other mental health professionals to become familiar with the language of spirituality within a healthcare context and the ways it enables meaning making and generates hope amidst some of the most challenging times in life (Swinton, 2001; Sims and Cook, 2009). Some authors argue that the current bio-psycho-social model is insufficient for the holistic care of people who use mental health services and call for a bio-psycho-social-spiritual model (Hefti, 2009). Acknowledging that religion and spirituality can be causing, mediating or moderating factors on mental health and can affect biological, psychological and social aspects of human life could assist clinical understandings of mental health difficulties as well as interventions which seek to meet an individual's holistic needs (Culliford and Eagger, 2009).

Understanding mental health difficulties in religious or spiritual terms offers an alternative to a biological or psychological framework and can assist the development of new perspectives, motivation and direction in life (Wong-McDonald, 2007). Russinova and Blanch (2007, p. 248) argue that ‘the successful incorporation of spiritual approaches into clinical practice has the potential to contribute to the next quantum leap in the development of effective person-centred systems of care’. Integrating a spiritual framework in mental healthcare practice could ‘open the door to a new and deeper vision of recovery – one that has long been espoused by consumer/survivors’ (Blanch, 2007, p. 255). Fallott (2007, p. 268) points out that the first task of mental health services is to become ‘spiritually informed’, building on our understanding of the roles that spirituality may play in mental health and recovery.

### Implications for practice

This review provides evidence about the importance of spirituality for many people with mental health difficulties and the roles it plays in their lives. These are primarily supportive but can also bring challenges particularly as people grapple to make meaning out of their experiences, often in isolation. Spirituality is core to many participants' identity and often reported as vital in helping them to cope with their distress and even preventing suicide. It is important therefore that mental health services and professionals are aware of and actively prepared to address and support this dimension, a recommendation which was made in nearly all of the reviewed studies (*n*  =  34). It is hoped that the MISTIC framework can support practitioners and others working in the field of mental health to do so and may be used to inform the development of spiritual assessment and interventions. To assist with the practical use and application of the MISTIC framework in clinical practice, Table 2 outlines clinical and practical considerations informed by the framework themes.

**Table 2.** Clinical considerations based on MISTIC framework

MISTIC theme pair	Clinical relevance	Clinical approaches and some potential questions
Meaning-making and Identity (MI)	**Understanding the centrality of spirituality** for some people's identities, lives and mental health. Spirituality is central to some people's identities and how they make sense of mental health difficulties and other experiences. Being open and actively exploring the important role spirituality may play in helping someone to make sense of their illness is a means of supporting recovery.	Being aware that people's spiritual experiences and beliefs may form a core part of their identity and their understanding of their mental health difficulties and/or recovery. Asking questions about the importance of spirituality in someone's life. Asking if religion or spirituality help them to make sense of what is happening and if so how. Asking if religion or spirituality are confusing and if so how.
Service provision and Talk about it (ST)	**Practical ways services can meet spiritual needs.** What services and practitioners can practically do to facilitate meeting people's spiritual needs and adopting an open approach towards communication about the topic.	Offering a pro-active yet sensitive approach in addressing the subject if someone would like this. Exploring people's spiritual needs whilst using services. Offering people the opportunity to talk about their spirituality and needs, and listening with sensitivity, empathy and open-mindedness. Listening non-judgmentally with care within a person-centred (rather than expert-driven and problem-solving) approach, giving the person space to express themselves, their own experiences and expertise.
Interaction with symptoms and Coping (IC)	**Awareness of challenges and coping strategies.** Understanding that spirituality can interact with mental health in complex and sometimes confusing ways and that people may employ a variety of spiritual practices and strategies to cope.	Exploring how people's spirituality and/or practices may be challenging or helpful. Asking about what spiritual and religious practices are used or have been used in the past. Asking questions about whether and how these practices are helpful or challenging. Asking questions about whether and how spirituality and religion help with coping. Asking questions about whether and how spirituality and religion lead to struggles or difficulties.

Spiritual assessment and care calls for similar clinical skills that are required for effective clinical practice generally, such as sensitivity, openness and empathy, but also require a thoughtful integration of the spiritual dimension of the person's life into treatment in a person-centred rather than a one-size-fits-all approach (Lomax and Pargament, 2016). These important considerations have led to an increasing literature around spiritual care, competence and assessment (e.g. Cook *et al*., 2009; McSherry and Ross, 2010; Eager and McSherry, 2011; Hodge, 2018) and the recommendation by The Royal College of Psychiatrists that patients' religious beliefs and spirituality should be sensitively explored and routinely considered as an essential component of clinical assessment (Cook, 2013).

### Study limitations and strengths

Qualitative systematic reviews are criticised for de-contextualising findings; however, to try to address this issue, Thomas and Harden's (2008) suggestions were followed including providing structured summaries of research contexts (online Supplementary material 3). Although the study aimed to create a simple way of framing complex information, such a strategy will always risk missing out important components. For example, studies with specific topics that were excluded from the review such as ‘specific religious/spiritual phenomena’ and ‘suicide’ may have provided important additional themes and insights relevant to or diverging from the MISTIC themes. These topics could be important ones to explore in future studies.

Issues of researcher interpretation were addressed through transparency and reflexivity, being systematic throughout the research and data analysis process, and striving to be as representative as possible of the research participants' accounts. A key study strength is that it provides new and important evidence about experiences of mental health and spirituality in a way in which is difficult with individual small-scale qualitative studies. The study spans a range of countries, cultures, religious and spiritual beliefs systems and mental health diagnoses, thus providing a diversity of contexts that contribute to the transferability and rigor of the findings. Finally, the MISTIC framework simplifies what can be a confusing and complex area of understanding for clinicians, and has the potential to impact on evidence-based training, interventions and policy guidelines.

### Conclusions and future developments

This study is the first qualitative systematic review to explore the experiences of spirituality among adults with mental health difficulties and revealed six key themes giving the acronym MISTIC. Future research is required to further refine the framework's applicability in clinical and training contexts and to create clear guidelines for this. The study offers a framework for developing holistic, strengths-focussed and person-centred approaches to mental health care, which have the potential to improve the quality of care and the experiences of people using mental health services.

## Supplementary material

For supplementary material accompanying this paper visit http://dx.doi.org/10.1017/S2045796019000234. click here to view supplementary material

## Availability of data and materials

The complete list of articles analysed in this review and supplementary materials are provided in the online Supplementary material.
