# Supplementary material for: The experiences of spirituality among adults with mental health difficulties: a qualitative systematic review
Source: Epidemiol Psychiatr Sci. 2019 May 3;29:e34. doi: 10.1017/S2045796019000234 (PMC8061134; doi:10.1017/S2045796019000234)
Supplement: Supplementary file 1 [file epssup.zip › S2045796019000234sup002.docx]

**Online Supplement 2: Medline search strategy (searched on 20/09/18)**

| **Medline**  Database: Ovid MEDLINE(R) and In-Process & Other Non-Indexed Citations <1946 to September 20, 2018>  Search Strategy:  --------------------------------------------------------------------------------  1 exp Mental Health/ (31669)  2 exp Mood Disorders/ (112171)  3 exp SCHIZOPHRENIA/ (98367)  4 exp Anxiety Disorders/ (74689)  5 exp DEPRESSION/ (103696)  6 exp Bipolar Disorder/ (37525)  7 exp Personality Disorders/ (39219)  8 exp Mental Disorders/ (1134218)  9 exp "BIPOLAR AND RELATED DISORDERS"/ (37533)  10 exp Neurotic Disorders/ (17936)  11 exp "trauma and stressor related disorders"/ (35801)  12 ("mental health problem*" or "mental health disorder*" or "mental disorder*" or "mental illness" or "mental health recovery" or "psychiatric disorder*" or "mental distress" or "emotional distress" or "mental health difficult*" or "mental health service user*" or "mental health patient*" or "mental health consumer*" or "mental health survivor*" or "psychiatric patient*" or "chronic mental illness" or psychosis or schizo* or "PTSD" or "spiritual crisis" or "spiritual emergency").ti,ab. (261875)  13 or/1-12 (1287750)  14 exp Spirituality/ (6621)  15 exp "RELIGION AND MEDICINE"/ or exp RELIGION/ or exp "RELIGION AND PSYCHOLOGY"/ (58866)  16 (spiritual* or religio* or faith or God or transcendent).ti,ab. (48577)  17 or/14-16 (86869)  18 13 and 17 (11224)  19 exp Personal Narratives/ (6876)  20 (experience* or "lived experience*" or belief* or view* or perception* or perspective* or attitude* or story or stories or narrative* or account* or "service user perspective*" or "patient perspective*" or "consumer perspective*").ti,ab. (2298991)  21 or/19-20 (2304706)  22 18 and 21 (4492)  23 exp Qualitative Research/ (41212)  24 exp INTERVIEW/ (28102)  25 exp Focus Groups/ (25202)  26 ("qualitative research" or interview* or "focus group*" or phenomenology or phenomenological).mp. (384775)  27 or/23-26 (384818)  28 22 and 27 (1150)  29 limit 28 to english language (1101)  *************************** |
| --- |
