# Supplementary material for: The experiences of spirituality among adults with mental health difficulties: a qualitative systematic review
Source: Epidemiol Psychiatr Sci. 2019 May 3;29:e34. doi: 10.1017/S2045796019000234 (PMC8061134; doi:10.1017/S2045796019000234)
Supplement: Supplementary file 1 [file epssup.zip › S2045796019000234sup003.docx]

**Online Supplement 3: Table of Characteristics of included studies (n=38)**

| **Study** | **Country** | **Participants** | **Religion or spirituality** | **Mental health issues** | **Design** | **Quality Appraisal rating** | **Key Findings** |
| --- | --- | --- | --- | --- | --- | --- | --- |
| 1. **Al-Solaim L, Loewenthal K** (2011). Religion and obsessive-compulsive disorder (OCD) among young Muslim women in Saudi Arabia. Mental Health, Religion & Culture **14**, 169-182. | Saudi Arabia | 15 female participants.  Arabic speaking, attending psychiatric clinics. | Muslim | Obsessive-compulsive disorder, onset before 21 years. | Semi-structured interviews.  Thematic analysis. | Medium | Participants turned first to faith-healers for help and preferred MHP to be religious. Religion and prayer were the main sources of coping with symptoms. Disruption of religious rituals caused by illness led to distress. |
| 2. **Baker M** (2010). How do service-users experience their local faith community and their mental health staff team? A UK perspective. Journal of Psychology and Christianity **29**, 240-252. | UK | 8 participants (2 male 6 female).  Recruited through UK national voluntary organisation for pastoral care and mental health. | Christian | All self-reported as having had previous though not current inpatient mental health admission in NHS. | Semi-structured interviews.  Interpretive Phenomenolog-ical Analysis. | Medium-high | People sought refuge or ‘sanctuary’ within religious and healthcare contexts. They often experienced restriction or themes of ‘imprisonment’. There was a lack of integration and understanding between faith and NHS contexts which impacted people’s experiences of recovery and meaning-making. |
| 3. **Buser J, Parkins R, Buser T** (2014). Thematic analysis of the intersection of spirituality and eating disorder symptoms. Journal of Addictions & Offender Counselling **35**, 97-113. | US | 12 participants  (all female).  Mean age 29.3 years.  Mostly white; Caucasian (n=11)  Middle-class (n=8).  Enrolled in mid-Atlantic Universities. | Range of religious affiliations:  Protestant, Catholic, atheist, agnostic, none. | Eating disorders:  All reported struggling currently (n=8) or in the past (n=4). | Semi-structured interviews.  Thematic analysis. | High | Spirituality imbues struggles with meaning, reduces symptoms and serves as an active agent for recovery. Spiritual uncertainty and feelings of guilt interacted negatively with symptoms. Participants shied away from requesting spiritual support. |
| 4. **Corry D, Tracey A, Lewis, C** (2015). Spirituality and creativity in coping, their association and transformative affect: A qualitative enquiry. Religions **6**, 499-526. | Ireland | 10 participants  (5 male 5 female).  Age 29 -70 years.  From Ireland (Republic and Northern).  Artists (n=3)  From contemplative prayer group (n=3)  Support group (n=4). | Catholic (n=9)  Of those:  2 identify as spiritual but not religious,  1 no denomination, engages in meditation. | Anxiety (n=6)  Depression (n=3)  Suicidal ideation (n=2)  Schizophrenia (n=2)  PTSD (n=1)  Bipolar disorder (n=1)  Eating disorder (n=1)  None (n=3) | Semi-structured interview.  Interpretive phenomenolog-ical analysis. | Medium | Spirituality is a very important and integral part of participants’ lives. They could not imagine life without it or cope without it. It played a role of positive transformation. Participants actively used spiritual beliefs to overcome symptoms, to enhance wellbeing and manage emotions/struggles. |
| 5. **Drinnan A, Lavender T** (2006). Deconstructing delusions: A qualitative study examining the relationship between religious beliefs and religious delusions. Mental Health, Religion & Culture **9**, 317-331. | UK | 7 participants  (6 male 1 female).  Age 30 – 53.  White (mostly English) (n=6)  Afro-Caribbean (n=1)  Recruited from community mental health teams. | Christian (n=3)  Mixed Christian (1 Jewish, 1 Hindu) (n=2)  ‘Idiosyncratic religious beliefs’ (n=2) | All experienced beliefs that had been diagnosed as delusional. | Semi-structured interviews.  Strauss & Corbin (1990) based qualitative methodology | Medium-high | Participants turned to religion to provide explanations for their psychotic experiences and negotiated identities which made sense of these. Religion had both positive effects (e.g. coping) and negative effects when interacting with symptoms. |
| 6. **Eltaiba N, Harries H** (2015). Reflections on recovery in mental health: Perspectives from a Muslim culture. Social Work in Health Care **54**, 725-737. | Jordan | 20 participants:  10 male (mean age 35),  10 female (mean age 34).  Jordanian/Palestinian backgrounds.  From Mental Health Centre in Jordan. | Muslim | Depression (n=7)  Anxiety (n=5)  Obsessive- compulsive disorders (n=4)  Panic attacks (n=4) | Semi-structured interviews. | Low | Religion was viewed as central to recovery and Allah as the main source of hope and understanding. Mental illness led to meaning in life and people’s relationship with Allah to become more prominent as they actively found ways to cope and find answers. |
| 7. **Hanevik H, Hestad KA, Lien L, Joa I, Larsen TK, Danbolt LJ** (2017). Religiousness in First-Episode Psychosis. Archive for the Psychology of Religion **39**, 139-164. | Norway | 18 participants  (12 male 6 female).  Recruited from Early Treatment and Identification of Psychosis Study, a clinical trial in Norway. | Most demonstrate religiousness at the present or in the past, majority engage in religious practices. Described subjectively for each participant. | First-episode psychosis | Semi-structured interviews and drawn images (though images not commented on in study).  Mixed-method. | High | Participants attempted to make sense of their hallucinatory experiences through religious explanations. Many participants used religiousness for coping. Some experienced negative religious coping and struggled with a crisis of meaning. |
| 8. **Heffernan S, Neil S, Thomas Y,**  **Weatherhead S** (2016). Religion in the recovery journey of individuals with experience of psychosis. Psychosis: Psychological, Social and Integrative Approaches **8**, 346-356. | UK | 10 participants  (8 male 2 female).  Age 25 – 35.  From Early Intervention Services, Community mental health teams and voluntary services across NW England | Muslim (n=4)  Christian (n=4)  Jehovah’s Witness (n=1)  Religious but no denomination (n=1) | Had experienced psychosis. | Interview with flexible schedule.  Social constructivist grounded theory. | High | Highlighted the essential role of a genuine reciprocal connection with a deity and an individually meaningful framework of understanding which assisted recovery (e.g. hope, self-esteem, emotional-wellbeing). Recovery was hindered when struggling with or lacking such a relationship. |
| 9. **Hustoft H, Hestad K, Lien L, Moller P, Danbolt L** (2013). "If I didn't have my faith I would have killed myself!": Spiritual coping in patients suffering from schizophrenia. International Journal for the Psychology of Religion **23**, 126-144. | Norway | 6 participants  (3 male 3 female).  Age 19 – 54.  Selected from an original sample (Danbolt et al., 2011) to examine different spiritual characteristics. | Mostly Christian backgrounds but differ in current understandings and conceptualisations of spirituality.  Believe in God (n=4)  Believe in a ‘higher power’ (n=2). | Diagnosed with broad schizophrenia spectrum disorders:  Paranoid schizophrenia (n=4)  Schizoaffective disorders (n=2). | Interviews.  Analysis with systematic text condensation. | Medium-high | Participants described their spirituality to have vital importance even if it met criteria for delusional. All showed spiritual struggles and attempts at spiritual coping. During illness they often searched to find new understanding. |
| 10. **Jones N, Kelly T, Shattell M** (2016). God in the brain: Experiencing psychosis in the postsecular United States. Transcultural Psychiatry **53**, 488-505. | US | 19 participants  (9 male 10 female).  Age 19 – 78.  Predominantly Caucasian (n=13)  African American (n=3)  Other (n=3).  High percentage advanced graduate degree or training. Recruited from fliers, clinical referrals and word of mouth. | Virtually all described childhood exposure to religion, majority identified as Christian. | Lifetime psychotic diagnosis, all reported at least one hospitalisation, past or present antipsychotic drug use, and repeated episodes of psychosis. | Unstructured interview with individually tailored questions for clarification.  Constructivist-grounded theory. | High | Participants drew on and integrated concepts from competing and often contradictory explanatory frameworks (e.g. clinical/scientific and spiritual.) Personal uncertainty and doubt was created by tensions between individual ideosynchratic experiences versus awareness of social and clinical doubt or scepticism. |
| 11. **Koslander T, Arvidsson B** (2007). Patients' conceptions of how the spiritual dimension is addressed in mental health care: a qualitative study. Journal of Advanced Nursing **57**, 597-604. | Sweden | 12 participants  (6 male 6 female).  Mean age 46 years.  Strategically selected from six wards at different psychiatric clinics in southern Sweden. | No information provided. | Depression (n=4)  Paranoid schizophrenia (n=3)  Psychosis (n=3)  Alcohol addiction (n=3) | Interview.  Phenomenology | Medium-high | Patients wished to have their spiritual needs addressed. Patients perceived nurses to avoid talking about spirituality and that they had insufficient knowledge about addressing spiritual needs. |
| 12. **Lilja A, DeMarinis V, Lehti A, Forssen A** (2016). Experiences and explanations of mental ill health in a group of devout Christians from the ethnic majority population in secular Sweden: a qualitative study. BMJ Open **6**, 1-9. | Sweden | 17 participants  (5 male 12 female).  Age 30 – 73 .  Mean age 50.  Purposive sampling within congregations in different parts of Sweden. | All with Christian denominational affiliation.  Lutherian Christian denominations (n=14). | Mixed range of mental health difficulties.  Included:  Depression (n=9)  PTSD (n=4)  Psychosis (n=3)  Bipolar disorder (n=2)  Panic disorder (n=2) | In-depth interviews.  Systematic text condensation analysis. | High | Participants’ relationship with God was often seen as the most important one in their lives, providing hope, protection, comfort and a life-line or prevention of suicide. Could also lead to disappointment, guilt, shame or fear of being unworthy.  Illness sometimes impeded ability to connect with faith/religion.  Explanations for illness included both medical and existential. |
| 13. **Macmin L, Foskett J** (2004). "Don't be afraid to tell." The spiritual and religious experience of mental health service users in Somerset. Mental Health, Religion & Culture **7**, 23-40. | UK | 27 participants  (10 male 17 female).  Age 20 – 70.  Mean age 45.  All white.  British (23). | Christian (past or present) (n=22):  Roman Catholic (n=7)  Anglican (n=5)  Methodist (n=5)  Baptist (n=3)  Quaker (n=2)  Spiritual not religious (n=7)  Pagan (n=6)  Buddhist (n=1) | Many with more than one diagnosis:  Depression (n=13)  Schizophrenia (n=6)  Manic depression (n=5)  Acute affective disorder (n=4)  Personality disorder (n=3) | Semi-structured interview.  Grounded theory approach.  Service-user lead research.  Based on larger scale ‘Somerset spirituality project’. | Medium-high | Vital importance that participants could safely explore their spiritual and religious needs and identify their spiritual resources in their search for meaning. Participants wanted to talk about spirituality to gain comfort from isolation and find meaning but struggled to reach out and were frustrated when ignored by MHPs. |
| 14. **Mahintorabi S, Jones MK, Harris LM** (2017). Exploring Professional Help Seeking in Practicing Muslim Women with Obsessive Compulsive Disorder Washing Subtype in Australia. Religions **8**, 137. | Australia | 5 female participants.  Age 33 – 45.  Not born in Australia – originally from Iran, Iraq, Turkey and Afghanistan.  Recruited via convenience sampling in Australian city. | Muslim | All had current or previous diagnosis of Obsessive-compulsive disorder and had washing compulsions for more than 1 hour per day. | Semi-structured interviews.  Thematic analysis within a ‘scientific realist framework’. | Medium-high | Religion both exacerbated symptoms (e.g. fear of sin and guilt) and provided a supportive coping mechanism (e.g. through prayer) to help people tolerate their symptoms. All participants sought help from an Imam before seeking help from a health professional which they found helpful and sometimes unhelpful. |
| 15. **Marsden P, Karagianni E, Morgan JF** (2007). Spirituality and clinical care in eating disorders: A qualitative study. International Journal of Eating Disorders **40**, 7-12. | UK | 10 female participants.  Recruited from eating disorder unit in Roehampton, UK. | All cited religion as important in questionnaire.  All Christian:  Roman Catholic (n=4)  Orthodox (n=1)  Congregational (n=1)  Salvation army (n=1)  Baptist (n=1)  Evangelical (n=1)  Pentecostal (n=1) | All diagnosed with DSM-IV anorexia nervosa (n=9) or bulimia nervosa (n=1). | Semi-structured ‘depth’ interviews. | Medium | Participants turned to their faith or new beliefs in a quest to resolve or understand difficulties.  Beliefs matured during treatment and people utilised practices such as prayer and being seen by a chaplain to explore meaning and for protection from suicide. People experienced spiritual struggles such as feelings of shame and sin. |
| 16**. Mental Health Foundation** (2002). Taken Seriously: The Somerset Spirituality Project. Mental Health Foundation: London. | UK | 25 participants  (10 male 17 female).  Age 20s to 70s.  Most in their 40s.  All white.  British (n=23).  All had been in contact with mental health services in Somerset.  Recruitment included posters, newsletters and through services. | Christian (past or present) (n=22):  Roman Catholic (n=7)  Anglican (n=5)  Methodist (n=5)  Baptist (n=3)  Quaker (n=2)  Spiritual not religious (n=7)  Pagan (n=6)  Buddhist (n=1) | All used mental health services for at least 6 months, most for over 10 years:  Depression (n=13)  Schizophrenia (n=6)  Manic depression (n=5)  Acute affective disorder (n=4)  Personality disorder (n=3) | Interviews.  Participant/ service user research. | Medium | Service users said they want spirituality to be taken seriously within mental health services and to be treated in a holistic way as a human being rather than an ‘illness’. Their spiritual journeys were times of personal discovery with ups and downs, confusion and insight, doubt and opportunities for transformation and healing. |
| 17. **Moller M** (1999). Meeting spiritual needs on an inpatient unit. Journal of Psychosocial Nursing & Mental Health Services **37**, 5-10. | US | 92 participants:  Patients (n=65) Family members (n=27)  Equally composed of men and women.  Age 19 – 81.  Recruited via newsletter and through a community health centre. | Catholic, Protestant,  Nondenominational Christian, Jewish,  Native American. Eastern religions including Islam and Buddhism. | Patients with psychiatric disabilities who had experienced inpatient hospitalisation. | 2 focus groups which became larger discussion groups due to large size. | Low | Four themes emerged in relation to spiritual needs participants identified during hospitalisation: comfort, companionship, conversation and consolidation.  Participants wanted reassurance from MHPs and were frustrated when they were labelled or unable to find someone who would listen. |
| 18. **Murphy M** (2000). Coping with the spiritual meaning of psychosis. Psychiatric Rehabilitation Journal **24**, 179-183. | US | 8 participants  (1 male 7 female).  Some attended psychosocial rehabilitation centre ‘clubhouse’. | Not specified - all saw faith as important to recovery. | All had experienced 1^ST^ psychotic episode between 7 – 35 years.  Schizophrenia (n=6)  Schizo-affective disorder (n=1)  Bipolar disorder (n=1). | Interviews.  Thematic analysis. | Low | Spiritual belief systems, attitudes and practices fostered attitudes that promoted health and wellbeing, empowered participants to make positive changes in their lives and provided sources of strength, faith, hope and coping. Some participants said it was their relationship with God/a higher power that allowed them to survive. |
| 19. **Nixon G, Hagen B, Peters T** (2010). Psychosis and transformation: A phenomenological inquiry. International Journal of Mental Health and Addiction **8**, 527-544. | Canada | 6 participants  (4 male 2 female).  Age 25 – 60.  Recruited via convenience sample and magazine advert.  Only considered if had transformational psychotic experience with higher level of functioning than when pre-psychotic. | All had a spiritual/ mystical aspect to their experience of illness. Most engaged in meditation, yoga, mindfulness and other spiritual practices (e.g. shamanic practices, healing, Buddhism). | All had psychotic experiences in the past with no episodes in last 5 years and not using psychotropic medication. | Narrative interviews.  Interpretive phenomenolog-ical approach. | High | All participants stated that finding and embracing a spiritual path was key to coping with their psychosis and integral to their transformation. They worked deliberately and determinedly at these paths and practices to achieve positive results. Highlighted ways in which psychosis can lead to positive changes such as personal growth, transformation, insight, healing and new career paths. |
| 20. **Ouwehand E, Wong K, Boeije H, Braam A** (2014). Revelation, delusion or disillusion: Subjective interpretation of religious and spiritual experiences in bipolar disorder. Mental Health, Religion & Culture **17**, 615-628. | The Netherlan-ds | 10 participants  (4 male 6 female).  Mean age 45.  All white Dutch and well educated.  All outpatients of ‘Altrecht’ mental health institution. Former clients of one researcher (n=4). | All raised in Christian tradition.  Christian (n=5):  Protestant (n=4)  Roman Catholic (n=1)  New Age Spirituality (n=4).  1 no religion but practiced Zen meditation | Bipolar disorder | Semi-structured interviews.  Phenomenolog-ical hermeneutic approach. | High | Nearly all participants experienced spirituality and religion and practices as a source of confidence, support and illness-management. Making sense of these experiences and considering their authenticity was an important issue but the spiritual and existential explanations exceeded the pathology. |
| 21. **Ouwehand E, Muthert H, Zock H, Boeije H, Braam A** (2018). Sweet delight and Endless Night: a qualitative exploration of ordinary and extraordinary religious and spiritual experiences in bipolar disorder. The International Journal for the Psychology of Religion **28**, 31-54. | The Netherlan-ds | 35 participants  (18 male 17 female).  Age 23 – 69.  Mean age 45.6.  Generally highly educated.  Recruited via mental health institutions, a peer support project and website/blog. | Most raised as Christian but changed spiritual attitude/beliefs.  ‘New spirituality’  (a contemporary personal expression of spirituality) (n=16)  Protestant (n=14) Roman Catholic (n=5)  Muslim (n=3)  Agnostic/Other (n=2) | Bipolar disorder 1 (n=27)  Bipolar disorder 2  (n=5)  Bipolar NOS (n=1)  Bipolar cycling (n=1)  Schizoaffective disorder (n=1) | Semi-structured interviews.  Phenomenolog-ical hermeneutic approach. | High | Participants reflected on their experiences in a wide variety of ways especially during manic episodes and in which language used often transcended medical terms. The most frequently reported feature of depressive episodes was absence of or distance from spirituality. |
| 22. **Oxhandler HK, Narendorf SC, Moffatt KM** (2018). Religion and spirituality among young adults with severe mental illness. Spirituality in Clinical Practice **5**, 188-200. | US | 34 participants from an original study of 55 participants (Narendorf et al. 2017) of the characteristics:  46% female.  Mean age 21.5.  African American 27%  White 27%  Hispanic 11%  Multiracial 11%  Recruited through a psychiatric unit. | Original study characteristics:  Christian (n=17)  Catholic (n=8)  Baptist (n=4)  Pentecostal (n=1)  Nondenominational (n=2)  Other (n=6)  None or unsure (n=16) | Participants had serious mental illness and had used crisis emergency services.  Presenting diagnosis of: bipolar disorder, major depressive disorder or a schizophrenia spectrum disorder. | From a previous mixed-methods study in which the topic of religion and spirituality was talked about unprompted.  Semi-structured interviews. | Medium-high | Four themes emerged: positive religious/spiritual coping, negative coping, relationship with God and implications for mental health. Religion/spirituality was described as a complex topic for this sample suggesting training is necessary to appropriately assess and integrate this area within health care. Suggestion for mental health providers to respect religious and spiritual diversity and demonstrate competence in this area of practice. |
| 23. **Raffay J, Wood E, Todd A** (2016). Service user views of spiritual and pastoral care (chaplaincy) in NHS mental health services: a co-produced constructivist grounded theory investigation. BMC Psychiatry **16**, 200. | UK | 22 participants  (17 male 5 female).  Median age 40 – 59.  Recruited from acute, medium and high secure in-patient services at NHS hospital. | Mostly Christian:  Roman Catholic (n=8)  Church of England (n=5)  Pentecostal (n=2)  United Reform (n=1)  Born-Again (n=1)  No denomination (n=1)  Multiple (n=1)  Atheist (n=1)  Did not identify (n=2) | Not specified.  Psychiatric inpatients. | Semi-structured interviews.  Co-produced research with service users.  Grounded theory and Symbolic interactionist approach. | High | A holistic approach and understanding of care which incorporates the spiritual dimension was suggested as important yet missing from conventional care. The importance of spiritual care and chaplaincy was highlighted as providing hope, someone who listens, normalising faith and assisting with recovery. |
| 24. **Rajakumar S, Jillings C, Osborne M, Tognazzini P** (2008). Spirituality and depression: The role of spirituality in the process of recovering from depression. Spirituality and health international **9**, 90-101. | Canada | 8 participants  (2 male 6 female).  From Canadian Mental Health and other associations and programmes. | Not specified.  All identified that spirituality played a role in their recovery. | Self-identified as having had depression. | Semi-structured in-depth interviews. | Medium-low | Spirituality played a significant role for participants in their recovery. This was primarily experienced as connection and relationship with God, a higher power, self, others and nature, and these provided meaning and purpose. |
| 25. **Rieben I, Mohr S, Borras L, Gillieron C, Brandt P, Perroud N, Huguelet P** (2013). A thematic analysis of delusion with religious contents in schizophrenia: open, closed, and mixed dynamics. Journal of Nervous & Mental Disease **201**, 665-673. | Switzerlan-d and Canada | 62 participants  (77% male 33% female).  Mean age 42.  White 92%.  From public psychiatric facilities in Geneva and Assertive Community treatment programme in Quebec. | Mostly Christian | All met ICD-10 criteria for  schizophrenia (82%) or schizoaffective disorder (18%).  Mean duration of illness 19 years with 9 hospitalisations. | Semi-structured interviews.  Content analysis and  Grounded theory. | Medium-high | Spiritual identities are constructed to give meaning to illness or as preferable identities to illness – these can be stable, single, plural, permeable or vulnerable to external influences.  Other important themes are finding meaning in the illness, spiritual figures and issues of guilt. |
| 26. **Rosli AN, Saini S, Nasrin N, Bahari R, Sharip S** (2016). 'I can't pray' - The spiritual needs of Malaysian Muslim patients suffering from depression. International Medical Journal Malaysia **15**. | Malaysia | 10 participants  (5 male 5 female).  Age 28 – 65 years.  Recruited from hospital database at in-patient psychiatric department. | Muslim | Patients who were previously diagnosed with major depressive disorder or persistent depressive disorder using DSM-5 criteria. | In-depth interviews. | Medium | Almost all participants expressed the need for worship. Knowledge and guidance were also important for people in these contexts, as well as existential needs such as calmness, hope, and meaning. |
| 27. **Russinova Z, Cash D** (2007). Personal perspectives about the meaning of religion and spirituality among persons with serious mental illness. Psychiatric Rehabilitation Journal **30**, 271 – 284. | US | 40 participants  (40% male 60% female).  Mean age 47.  Caucasian 90%.  Recruited from larger study on alternative medicine and recovery. | All experienced healing benefits from at least one and average 5 alternative healing practices e.g. meditation, massage, yoga and prayer.  Christian (n=23)  Eastern tradition (n=5)  Other or none (n=12)  All ‘very spiritual’.  Not or slightly religious (56%). | Self-identified as having serious mental illness:  Bipolar disorder (n=17)  Schizophrenia spectrum disorder (n=13)  Depressive disorder (n=9)  Other (n=1) | In-depth semi-structured telephone interviews.  Part of larger mixed-methods study. | Medium | Participants had deep yet finely nuanced understandings of concepts of religion and spirituality, which were rich in scope. They had distinct contrasting understandings of religion and spirituality. The study highlighted the multi-dimensionality of the concepts with a diversity of meanings.  Religion was often seen as more prescriptive whilst spirituality as more explorative and intrinsic. |
| 28. **Salimena A, Ferrugini R, Melo M, Amorim T** (2016). Understanding spirituality from the perspective of patients with mental disorders: contributions to nursing care. Revista Gaucha de Enfermagem **37**. | Brazil | 9 participants.  Male and female but numbers not specified.  Age 35 – 64.  From a psychosocial care centre in the city of Minas Gerais in Brazil. | Not specified (though language from quotations suggests predominantly Christian and Catholic). | Schizophrenia, Bipolar disorder, Panic attacks,  Depression,  Numbers not specified. | Open-ended interview.  Phenomenology | Low | Religion helped the participants cope with their illness and brought encouragement, strength, meaning, and positive change. Attending church was important for people to express their faith. |
| 29. **Smith S, Suto M** (2012). Religious and/or spiritual practices: extending spiritual freedom to people with schizophrenia. Canadian Journal of Occupational Therapy - Revue Canadienne d Ergotherapie **79**, 77-85. | Canada | 9 participants  (5 male 4 female).  Age 39 – 59.  From community mental health teams in the Vancouver area. | Could clearly articulate their ideas about spirituality.  Diverse viewpoints along a continuum (e.g. religious, theistic, mystical, existential). | Diagnosis of schizophrenia confirmed by mental health professional. Not had hospital admission within 6 months. | Interviews.  Combination of symbolic interactionism, hermeneutics and phenomenology | High | Engagement in religious and spiritual practices gave participants a way to find meaning, experience empowerment and cope with mental health difficulties. Participants valued agency, freedom and spiritual choice. |
| 30. **Smith S, Suto M** (2014). Spirituality in bedlam: exploring patient conversations on acute psychiatric units. Canadian Journal of Occupational Therapy - Revue Canadienne d Ergotherapie **81**, 8-17. | Canada | 7 participants  (5 male 2 female).  Age early 20s – late 60s.  Patients recruited from acute psychiatric unit via patient meeting who were not at the time experiencing psychosis. | Diverse expressions:  Christian (n=2)  Spiritual but not religious (n=2)  Mormon (n=1)  Not defined but engage in spiritual practices (n=2). | Not specified.  Admitted to acute psychiatric unit at time of study for at least 3 to 4 days, apsychotic and able to reflect. | Focus groups and interviews.  Community-based participatory research. Interpretive description. | High | Participants desired authentic spiritual conversations in mental healthcare contexts but were aware of MHPs caution and propensity to judge or classify their spirituality negatively. They wanted them to ‘just ask the question’. |
| 31. **Sreevani R, Reddemma K** (2012). Depression and spirituality - a qualitative approach. International Journal of Nursing Education **4**, 90-93. | India | 8 participants  (2 male 6 female).  Age 20s – 40s.  Recruited by referral from psychiatrist using purposive sampling in psychiatric outpatient department. | Hindu (n=6)  Muslim (n=1)  Christian (n=1) | All diagnosed with mild to moderate depression based on ICD 10 criteria. | Semi-structured interview.  Qualitative descriptive approach. | Low | Spiritual practices and activities were important to all participants and helped them control negative thoughts, fear and tension. They also had faith God would help them. The illness and symptoms at times impeded their ability to perform rituals. |
| 32. **Starnino V** (2014). Strategies for incorporating spirituality as part of recovery-oriented practice: Highlighting the voices of those with a lived experience. Families in Society **95**, 122-130. | US | 18 participants  (6 male 12 female).  Age 20 – 62.  Mean age 43.  White (n=10) Native American (n=3)  African American (n=2)  Mixed race (n=3) Using services at a Midwestern mental health centre. | Spiritual but not religious (n=8)  Christian (n=7)  Native American spirituality (n=2).  1 ‘exploring’. | Psychotic disorder (n=7)  Bipolar disorder (n=6)  Major depressive disorder (n=4)  Dissociative identity disorder (n=1) | Two face-to-face interviews.  Hermeneutic phenomenology | High | Participants found it very helpful when MHPs expressed a genuine curiosity and interest in their spirituality and showed a willingness to discuss ways they use beliefs and practices. Effective listening and a non-confrontational approach were found to be particularly helpful whilst challenging their spiritual experiences was unhelpful. |
| 33. **Starnino V, Canda E** (2014). The spiritual developmental process for people in recovery from severe mental illness. Journal of Religion & Spirituality in Social Work: Social Thought **33**, 274-299.  (Larger study from which above study was derived). | US | 18 participants  (6 male 12 female).  Age 20 – 62.  Mean age 43.  White (n=10) Native American (n=3)  African American (n=2)  Mixed race (n=3).  Using services at a Midwestern mental health centre. | Spiritual but not religious (n=8)  Christian (n=7)  Native American spirituality (n=2).  1 ‘exploring’. | Psychotic disorder (n=7)  Bipolar disorder (n=6)  Major depressive disorder (n=4)  Dissociative identity disorder (n=1) | Two face-to-face interviews.  Hermeneutic phenomenology | High | Participants’ experiences differed in relation to levels of spiritual development and the extent to which they were able to integrate their spirituality and recovery.  This was described as a non-linear and lengthy process of which spiritual benefits (e.g. meaning-making and improved self-concept) and struggles (e.g. interaction of symptoms) are a natural part. |
| 34. **Starnino V** (2016). Conceptualizing spirituality and religion for mental health practice: Perspectives of consumers with serious mental illness. Families in Society: The Journal of Contemporary Social Services **97**, 295-304. | US | 18 participants  (6 male 12 female)  Age 20 – 62  (Details as above – subsection of above study). | Spiritual but not religious (n=8)  Christian (n=7)  Native American spirituality (n=2).  1 ‘exploring’. | Psychotic disorder (n=7)  Bipolar disorder (n=6)  Major depressive disorder (n=4)  Dissociative identity disorder (n=1) | Two face-to-face interviews.  Hermeneutic phenomenology | Medium-high | There were important nuances, and much variation and overlap in relation to how people with mental health difficulties defined and conceptualised spirituality and religion. Many said they did not want to be constrained by someone else’s definition of these concepts. |
| 35. **Sullivan W** (1993). "It helps me to be a whole person": The role of spirituality among the mentally challenged. Psychosocial Rehabilitation Journal **16**, 125-134. | US | 19 participants.  From an original study of 40 participants of the characteristics:  48% male, 52% female.  Mean age 39.  Former or current consumer of mental health services. | Not specified. | Of original sample of 40:  Schizophrenia (75%)  Bipolar disorder (20%)  Other (5%)  Mean number of hospitalisations 5.6 | Open-ended interview.  Retrospective analysis. | Low | Spirituality was identified from a larger study as the most commonly mentioned factor for success. Spiritual beliefs and practices were important, in particular people’s relationship with God or a spiritual presence which could provide comfort, guidance and help with coping. |
| 36. **Wilding C, May E, Muir-Cochrane E** (2005). Experience of spirituality, mental illness and occupation: A life-sustaining phenomenon. Australian Occupational Therapy Journal **52**, 2-9. | Australia | 6 participants  (3 male 3 female).  Age 35 – 55.  Recruited through two rural Australian community mental health centres. | Not specified. | Diagnostic information not obtained however some self-reported:  Depression (n=4)  Bipolar disorder (n=1)  Psychosis (n=1)  Anxiety disorder (n=1) | In-depth interviews.  Phenomenology: Heidegger’s (1962) hermeneutic circular approach. | High | Spirituality was vitally important for participants and it sustained and enhanced their lives. It did this by providing meaning, sustaining mental and emotional wellbeing and by preventing suicide. Spirituality was a core aspect of people’s identity and sense of self. |
| 37. **Wilding C, Muir-Cochrane E, May E** (2006). Treading lightly: spirituality issues in mental health nursing. International Journal of Mental Health Nursing **15**, 144-152.  (Same study as above with different findings reported.) | Australia | 6 participants  (3 male 3 female).  Age 35 – 55.  Recruited through two rural Australian community mental health centres | Not specified. | Diagnostic information not obtained however some self-reported:  Depression (n=4)  Bipolar disorder (n=1)  Psychosis (n=1)  Anxiety disorder (n=1) | In-depth interviews.  Phenomenology :Heidegger’s (1962) hermeneutic circular approach. | High | Participants defined and experienced spirituality in unique ways and as a life-long journey that changes over time and can be personally transformational.  Often mental illness instigated the spiritual journey and was perceived as useful or having meaning because of this. Participants had a strong desire to talk about and share their spiritual experiences but often encountered difficulties in doing so with MHPs. |
| 38. **Yang C, Narayanasamy A, Chang S** (2012). Transcultural spirituality: the spiritual journey of hospitalized patients with schizophrenia in Taiwan. Journal of Advanced Nursing **68**, 358-367. | Taiwan, China | 22 participants  (10 male 12 female)  Age 29 – 63 years.  Mean age 42 years.  From 2 psychiatric hospital long-term rehabilitation units. | Buddhist (n=7)  Catholic (n=5)  Multi-religion believers (Buddhism and Taoism) (n=4)  Atheist (n=6) | All diagnosed with schizophrenia by IDC-10 but not experiencing acute problems at time of study. | Semi-structured interviews.  Thematic analysis. | Medium-low | Participants expressed spiritual distress as a result of prolonged hospitalisation (e.g. struggle with self-worth, confusion, loss of hope). They often made sense of what was happening in relation to Chinese philosophy e.g. that illness was predetermined by fate assigned by a higher power, was due to karma and that suffering was a spiritual practice and test. |

Key

MHPs = Mental Health Professionals
