# Supplementary material for: The experiences of spirituality among adults with mental health difficulties: a qualitative systematic review
Source: Epidemiol Psychiatr Sci. 2019 May 3;29:e34. doi: 10.1017/S2045796019000234 (PMC8061134; doi:10.1017/S2045796019000234)
Supplement: Supplementary file 1 [file epssup.zip › S2045796019000234sup004.docx]

# Online Supplement 4 – Detailed Theme Descriptions

# Description of Themes

The six main themes are now described along with their sub-themes. Although each one was distinct there was overlap between them all, in particular between consecutive pairs of themes, Meaning-making and Identity (MI), Service provision and Talk about it (ST) and Interaction with symptoms and Coping (IC) which provide some clinical applications as outlined in Table 2.

## 1. Meaning-making

The theme of Meaning-making refers to the ways in which people made sense of or struggled to make sense of their experiences of mental illness and how these interpretations could often change over time. This was one of the most frequently occurring themes and was mentioned throughout nearly all (33) of the studies. This is perhaps unsurprising as meaning is seen as central to most definitions of spirituality. This theme also contained three sub-themes, Multiple explanations, Developmental journey and Destiny versus autonomy.

Many studies highlighted that participants provided explanations of illness that were related to their spiritual or religious beliefs which, in turn would give meaning to their suffering or to specific symptoms (Rieben et al., 2013; Lilja et al. 2017; Oxhandler et al., 2018). Sometimes this took the form of an ‘overflow of meaning’ in which participants during manic episodes felt a heightened sense of purpose, ‘synchronicity’ or ‘divine direction’ (Ouwehand et al., 2018: 46). One of the central components to this theme was the idea of a quest or search for meaning (Macmin & Foskett, 2006). Ouwehand et al. (2014) describe how the quest for meaning for the participants with bipolar disorder in their study sometimes began after a religious experience they had during initial or subsequent manic episodes. They would then engage in attempts to make these experiences comprehensible. However, participants often questioned the authenticity of their religious experiences, doubting how to interpret and how much weight to give them.

Such confusions, doubts and struggles within the meaning-making process were very common amongst participants across studies, sometimes resulting in a ‘crisis of meaning’ (Hanevik et al., 2017). This was often because of the conflicting accounts that people were exposed to regarding their mental health and spiritual experiences.

### 1.1 Multiple explanations

Heffernan et al. (2016) describe how participants questioned the origin of their experiences of psychosis by considering multiple explanations. Because of the conflicting views participants received as a result of the lack of integration of mental health services and religious organisations (Baker, 2010) they often found it very difficult to arrive at an explanatory framework. This framework was considered key because ‘when you don’t know, it’s harder to deal with. When you know it’s a lot easier to deal with’ (Heffernan et al. 2016: 352). Recovery could be impeded when participants lacked a structure for understanding their experiences or remained confused between competing models of explanations.

Sometimes people successfully used concepts from different linguistic fields or from medical and spiritual perspectives simultaneously (Ouwehand et al., 2014) developing ‘multicausal models of explanation’ which were not experienced as incompatible. For example, Jones et al. (2016) found that participants juxtaposed and blended constructs associated with bio-psychiatry and ‘consensus reality’ alongside those associated with religious or spiritual, paranormal or magical themes. However, participants were also often acutely aware of ways in which their experiences and interpretations might be judged by others.

Despite these doubts and tensions between different explanatory frameworks, Smith & Suto (2012) found that the participants in their study became quite adept at utilising their spiritual discourse to empower them and meet their unique needs. Perhaps because they had to grapple with such doubts and tensions, they showed a ‘tremendous ability to dialogue between different systems of meaning’ (Smith & Suto, 2012: 82). The degree to which people were able to do this however often depended on how people made sense of these experiences over time.

### 1.2 Developmental journey

Many of the studies reported that participants’ perceptions about and relationships with their spirituality were dynamic over time (Lilja et al., 2017), highlighting a change in meanings or a journey of inner development (Baker, 2010). Participants in Buser et al.’s (2014) study described movement from spiritual uncertainty to spiritual commitment and confidence during their recovery, experiencing a decrease in symptoms as they did so. Others reported a change in the ways in which they experienced, valued and expressed their spirituality (Wilding et al., 2006). For example, Marsden et al. (2007) found that participants in their study experienced a maturation of their religious beliefs or converted to a new faith in a quest to resolve or understand difficulties.

Sometimes people’s mental health difficulties would act as a ‘call’ to participants’ spiritual life because it was not until then that spirituality became vitally important to them. As one participant in Wilding et al.’s (2006:147) study stated, ‘One of the things that I have actually gained from having a mental illness [is] that I…have looked at what this God stuff means.’ Another participant in the same study remarked, ‘…if I hadn’t gone mental I wouldn’t have gone spiritual.’

Many participants viewed their spirituality as a path or journey involving phases of ‘dipping in’ and ‘sitting back’, ups and downs, periods of confusion and doubts, as well as insights and opportunities for transformation (Mental Health Foundation, 2002). One participant said she preferred to conceptualise her experience of psychosis as a process of spiritual transformation rather than a psychiatric disability:

‘I tend to err on the side of it being a transformational process. So that I can work with it. Because if it’s a mental illness, it shuts me down’ (Nixon et al., 2010: 538).

Participants frequently described how their experiences of illness had changed their lives in positive ways (Corry et al., 2015). They sometimes described their illness as a gift from God because it allowed them to heal from past traumas or personal issues (Rieben et al. 2013) or become better, stronger and more empathetic people as a result (Murphy, 2000).

Sometimes this transformation involved the development of new spiritual beliefs. If people were left to interpret their experiences in solitude there was a greater chance the belief systems could be interpreted as delusional (Hustoft et al., 2013). However, when people had supportive people to explore the meaning-making process with, this provided opportunities for their own spiritual resources to emerge and their ‘breakdowns’ to ‘break-throughs’ (Macmin & Foskett, 2004).

In a comprehensive analysis of the ways in which participants with severe mental illnesses utilised spirituality as part of their spiritual development, Starnino & Canda (2014) found that they demonstrated a potential to achieve a positive integration of their spirituality and recovery. However, this could be a complex, lengthy and non-linear process, requiring focus and dedication towards their spiritual development. They also found that participants who were able to renew their spirituality and had more helpful and affirming worldviews were able to reach higher levels of integration than those who had self-denigrating spiritual beliefs.

### 1.3 Destiny versus autonomy

A final subcategory within the over-arching theme of Meaning-making concerned the level of choice and control people conceptualised themselves as having in relation to their religious or spiritual belief systems. On one end of this spectrum was the sense that what was happening was divinely intentioned, often involving a sense of fate, destiny or as being subject to ‘God’s will’. For example, many of the Muslim participants in Eltaiba & Harries’s (2015) study conceptualised what was happening to them as a trial sent by Allah and part of their ‘Qadar’ or destiny:

‘The illness is Allah’s will … It is a trial … I pray to Allah every day to make me feel better’ (Eltaiba & Harries’s, 2015: 732).

Attributing the cause of illness to an external spiritual force was also significant in some of the other non-western studies. For example, participants in Yang et al.’s (2011) study viewed mental illness in relation to beliefs influenced by Chinese philosophy such as Taoism and Buddhism. Here, mental illness was sometimes understood as an unpredictable difficulty in an individual’s ‘Ming’ or fate, assigned by ‘Tien’ or a higher being:

‘Ming arranges our lives. If I have to stay in the hospital and suffer from this illness, that is my Ming’ (Yang et al., 2011: 361).

The idea of ‘God’s will’ was important for some Christian participants, viewing everything that happened in life as an expression of God’s will in action (Nixon et al., 2010). Some people conceptualised this sense of destiny more as a letting go of control or of surrender. Being able to rely on a presence greater than oneself and perceiving that one didn’t have to know all the answers was very reassuring to some people (Wilding et al., 2005; Corry et al., 2015).

On the other hand, a sense of choice and control, agency and freedom were important for many participants, sometimes expressed through the ways they conceptualised and cultivated their own unique spiritual belief systems and identities and which provided a sense of autonomy and empowerment (Smith & Suto, 2012; Heffernan et al., 2016):

‘…it should be up to that person to have that choice… if you have belief in yourself and believe what you believe then it’s not put into you… it’s not forced’ (Heffernan et al., 2016: 350).

Participants made sense of their experiences in a variety of often complex ways involving multiple and sometimes contradictory explanatory frameworks which changed over time and included struggle and growth. They might view what was happening as part of their destiny or as an opportunity to develop a sense of autonomy within their meaning-making processes.

The second main theme is connected with the theme of Meaning-making and involves the centrality of spirituality to many people’s lives and their identities.

## 2. Identity

Identity was a prominent theme (mentioned in 20 studies) and refers to the centrality of spirituality for many people’s lives and core sense of self. Spirituality represented for many the core essence of who they are, shaping their identities through their experiences of illness, struggle, recovery and meaning-making.

People’s identities related to how they understood their religion or spirituality. Although people sometimes saw religion and spirituality as intertwined (Salimena et al., 2016), they more often wanted to distinguish between spirituality and religion, especially those who identified as being spiritual but not religious (Corry et al., 2015). Some participants viewed religion as more structured, prescriptive and extrinsic whilst spirituality was ‘inner and personal’ (Moller, 1999: 6), exploratory (Russinova & Cash, 2007) and ‘not constrained by anyone else’s definition’ (Starnino, 2016: 300). Wilding et al., (2006) found that it was important for people to define spirituality in their own unique ways and that no participant had exactly the same configuration of religious or spiritual beliefs.

Participants drew on their spiritual frameworks to develop and negotiate a spiritual identity as a ‘spiritual being’ rather than that of a ‘patient’ as suggested by diagnostic labels (Drinnan & Lavender, 2006 Wilding et al., 2006). Spirituality was seen as vital to life and enabled many participants to develop a healthier more empowered view of themselves, recognising that they were ‘good enough’ despite their illness (Wilding et al., 2006; Heffernan et al., 2016; Starnino & Canda’s, 2014).

Sometimes people’s beliefs helped them to feel special (Heffernan et al., 2016) or as if they had special abilities or powers, and these could help them to cope during times of stress or anxiety (Jones et al., 2016). Mystical experiences could also affect how people saw themselves, such as a sense of unity (Ouwehand et al., 2014) or a sense of transcending the self (Nixon et al., 2010). These kinds of experiences could be pleasant and helpful or distressing and confusing, for example feeling a disruption from a singular sense of self, sometimes leading to a feeling of being possessed or controlled by another entity (Rieben et al., 2013). These more unusual experiences often arose as a result of the ways in which people’s mental health symptoms and spirituality interacted, which is described in further detail below. Although sometimes bizarre or disturbing, they were still considered to be core to how people understood themselves:

‘…to invalidate a person’s spirituality no matter how distorted that is, is to invalidate that real core sense of self and I think once you do that you risk doing untold damage to somebody’ (Mental Health Foundation, 2002:22).

It was important for many to feel they could be accepted for who they are especially because of their experiences of being labelled and rejected due to their mental health problems (Mental Health Foundation, 2002). Whether this was possible within the context of mental health services is the subject of the next key theme.

## 3. Service provision

The theme of Service provision was mentioned in 23 studies and relates to the ways in which people’s spiritual needs are addressed or not within mental health care services and how people described their interactions with services and mental health professionals.

The most commonly recurring experience under this theme was that participants felt their religious experiences were often dismissed, misunderstood, pathologised and taken as confirmation that they were mentally ill. This often resulted in frustration at the lack of opportunities within services to explore the meaning of their experiences (Drinnan & Lavender, 2006; Jones et al., 2016). Because this was a very common experience, a key concern about service provision and seemed an important theme to highlight in its own right within a framework for clinicians, it is described under a separate theme Talk about it.

Participants also reported a lack of facilities within services (Heffernan et al., 2016), a lack of opportunities to explore their needs (Yang et al., 2011) and a lack of provision for spiritual practices (Salimena et al., 2016) despite wanting their spiritual needs addressed within these contexts (Koslander & Arvidsson, 2007).

One of the reasons it was so important for participants to have their spiritual needs addressed within mental healthcare settings was because these could be the most challenging times of their lives and they craved safety, familiarity and reassurance during their frequently distressing encounters with services:

‘I felt very alone and isolated in a strange environment, one which I hadn’t experienced before and things were happening to me that I didn’t know… I wanted some kind of stability within that and that was why my faith and religion were coming in at that time … I wanted to identify with it as soon as possible’ (Mental Health Foundation, 2002: 20).

People talked about ways in which mental health professionals and services could provide for their spiritual needs. In a study by Nabil et al. (2016) Muslim participants suffering from depression in Malaysia identified two distinct sets of spiritual needs, one relating to their religious practices such as worship, religious knowledge and guidance, and the other which was referred to as ‘existential’, such as the need for calmness and for sensitivity and empathy from practitioners. Some participants highlighted the importance of being given access to safe and quiet spaces where they could engage in spiritual practices (Mental Health Foundation, 2002). They spoke of the desire to explore practices such as meditation and yoga or breathing exercises (Sreevani & Reddemma, 2012) and of the usefulness of a café-style menu of options giving examples of spiritual resources on offer (Smith & Suto, 2014). Participants also suggested that practitioners could facilitate involvement with spiritual communities or offer directive strategies such as recommending spiritual readings (Starnino, 2014; Sreevani & Reddemma, 2012).

Participants were aware of the difficulties and demands this kind of care could place upon mental health professionals; that it could be ‘unknown territory’ or even ‘threatening’ for some (Mental Health Foundation, 2002: 25). Some acknowledged that to be able to offer this kind of care staff also require opportunities to care for themselves psychologically and spiritually. Alternatively, these needs could be met through spiritual care services including chaplaincy which were seen by some as essential for recovery and providing hope as they normalised faith and provided a listening ear without an agenda:

‘[spiritual care’s] very very important for mental health; sometimes it’s the only thing that seems, that can maybe get through to someone. It’s a different level of understanding, that goes beyond words, that goes beyond, something you can touch, it goes beyond all that…’ (Raffay, Wood & Todd, 2016: 5)

When health care professionals did consider the spiritual needs of the participants this could make a great deal of positive impact on their lives and recovery. The most important ways in which they could do this was by proving opportunities to talk about it.

## 4. Talk about it

The theme Talk about it interconnects with the theme of service-provision and refers to the ways in which practitioners could most helpfully support people’s spiritual needs as well as what participants across 13 studies said they often needed most generally in relation to their experiences of spirituality and mental health.

One of the greatest challenges that participants struggled with during the meaning-making process is that that they were often forced to negotiate their experiences in relative social and cultural isolation (Jones et al., 2016). They wanted to talk to gain comfort from the trauma and loneliness of their distress and to understand the meaning of their ill-health in religious and spiritual terms (Mental Health Foundation, 2002; Macmin & Foskett, 2004). They looked to healthcare staff to help them with this because the experiences could be extremely difficult to interpret alone and doing so could lead to confusion or have adverse effects on recovery (Ouwehand et al., 2014; Heffernan et al., 2016)

Although nearly all participants in Ouwehand et al.’s (2014) study expressed an explicit wish and strong desire to talk about religion and spirituality during treatment, they had concerns about the difficulties in doing so with mental health professionals. Key themes that arose were worry about whether their beliefs and experiences would be accepted by healthcare professionals and fear of being labelled mentally unwell when their spiritual experiences included events that could be interpreted as symptoms of mental illness (Wilding et al., 2006). Some participants felt huge frustration at their inability to find staff who would just listen to them (Moller, 1999) or when staff ignored their spirituality, treating it only as signs of illness (Macmin & Foskett, 2004). When healthcare practitioners challenged the legitimacy of spiritual experiences, this was described by one person as devastating:

‘Many people don’t realise how devastating it is to be told that that’s not real, that that’s fantasy … Because to the people it is real and it needs to be treated as if it is real instead of just discarded and pushed aside, because it is a very big part of people … It’s their core’ (Starnino, 2014: 127).

Some participants lacked confidence in practitioners’ ability to bring up the subject of spirituality, noticing their avoidance of the issue (Koslander & Arvidson, 2006; Murphy, 2000). The process of engaging in conversations with staff was sometimes a cautious one, as participants ascertained whether it felt safe to talk about spirituality with staff, feeling like ‘conversational eggshells’ because of the risk entailed in becoming vulnerable by exposing their personal spiritual beliefs (Smith & Suto, 2014: 13; Smith & Suto, 2012). Others said that they felt reluctant to talk to professionals at all as they had received negative judgments in the past:

‘I don’t tell people because they don’t believe ya, you know, so it’s not worth it’ (Wilding et al., 2006: 148).

Baker (2010: 249) found that service users in his study developed skills of non-disclosure and self-control as a form of self-protection within a ‘strategy of silence’. One participant in Wilding et al.’s (2006: 148) study said that he felt that spirituality was a ‘taboo subject’ but if mental health practitioners were to be holistic, as he felt many claimed to be, they really should discuss spirituality with their clients.

When asked what is helpful within these contexts, participants were often quite clear:

‘Just ask the question!’ (Smith & Suto, 2014: 13).

‘Really listen with your personal attention’ (Moller, 1999: 9).

‘Listen to what the person has to say … whether it’s good or bad, bizarre or whatever’ (Starnino, 2014: 126).

‘Please reassure me that I will get through this’ (Moller, 1999: 9).

As well as the importance of asking, listening and reassurance, participants also said it was helpful that practitioners bring up the subject on their own initiative (Ouwehand et al., 2014; Koslander & Arvidsson, 2006).

When participants could speak openly with practitioners they usually found this very helpful and were very appreciative of the efforts staff went to in order to sensitively approach the subject and listen to their spiritual experiences, including those that seemed unfamiliar or different to them (Starnino, 2014). A participant in one study reflected that he felt closer and more trusting of his psychiatrist after having a conversation about his Christian faith (Smith & Suto, 2014). Another recounted how a nurse had helped by asking him pertinent questions:

‘The community psychiatric nurse was terrific. Although he was not a Christian, he asked me very, very pertinent questions about how I could reconcile my faith with what was happening to me and what God meant to me’ (Mental Health Foundation, 2002: 23).

The final two key themes move from service context to a focus on coping with the difficulties of mental health problems and how spirituality interacts with these experiences.

## 5. Interactions with symptoms

Interaction with symptoms was a theme addressed by 18 studies. This theme describes how people’s symptoms or mental health difficulties interacted with their spirituality, often in quite challenging or disruptive ways. This was the most complex theme and was rarely well defined or developed within the studies, perhaps because it lacks clarity as a topic generally. Two distinctive features could be delineated about it however. Firstly, Interactive meaning-making, describes the ways in which the interaction between spirituality and mental health symptoms were connected with unusual experiences and the attempts to make meaning from these experiences. Secondly, Spiritual disruption describes how mental health symptoms could disrupt people’s ability to engage in spirituality.

### 5.1 Interactive meaning-making

The first sub-theme links to the theme Meaning-making and the sub-theme Multiple explanations as this experience could often involve a clash of multiple realities where people would be left somewhere ‘in a state of being betwixt and between that had its own particular features’ (Jones et al., 2016: 496). For half of the participants in Starnino & Canda’s (2014) study this involved experiencing visions, hearing voices from nonphysical entities or having beliefs which included spiritual or religious concerns or ideas. These experiences were classified by Drinnan & Lavender (2006) under a theme of ‘unusual religious experiences’ which included persecutory or grandiose religious beliefs. Sometimes people’s spirituality intersected with their symptoms, for example in Buser et al.’s (2014) study of people with eating disorders, participants sometimes perceived their symptoms as a form of gluttony that was ‘sinful’ in their spiritual tradition.

Starnino & Canda (2014) suggested that a key factor determining how people experienced these interactions was how effective or convincing their belief systems were at providing explanations for their unusual experiences or whether they provided a sense of hope. The close weaving together of spirituality and symptoms therefore may have functioned as a way of meaning-making in a world of changed perception (Hustoff et al., 2013). Hanevik et al. (2017) found that one of the main characteristics of religiousness for their study participants with first-episode psychosis was that it provided a mystical explanation for hallucinatory experiences. By providing meaning to their psychotic experiences, this helped them to gain a sense of control and coherence in their lives. Other ways participants might interpret their hallucinatory or mystical experiences included feeling that they were on a ‘sacred mission’ to save the world (Hanevik et al., 2017), feeling a strong sense of altered awareness as if taken over by another reality (Ouwehand et al., 2018) or that they had supernatural powers or extraordinary spiritual gifts (Hustoft et al., 2013).

### 5.2 Spiritual disruption

The second sub-theme, Spiritual disruption refers to the ways in which mental health symptoms could impede people’s ability to connect with their spirituality and engage in spiritual practices. For example, performing religious rituals was considered highly important for the Muslim participants in Al-Solaim & Loewenthal’s (2011) study and the disruption to these practices caused by their symptoms triggered high levels of guilt and anxiety. Some participants reported reductions in ability to focus, problem-solve and think abstractly when in a state of psychosis, which resulted in difficulties connecting with spiritual practices and rituals (Moller, 1999; Heffernan et al., 2016; Sreevani & Reddemma, 2012). Others struggled to attend church services because they felt too tired, ashamed or that they no longer fitted in. This could result in losses in the ways they could express their faith and fears that their most important relationship in life was broken (Lilja et al., 2016).

Despite these difficulties, some participants stressed that their experiences had spiritual or existential implications that exceeded the pathology (Ouwehand et al., 2014). They maintained the belief that there was something about their experiences that was more than, beyond and not wholly explained by illness (Starnino & Canda, 2014).

The best ways for practitioners to approach these scenarios, participants suggested, was a gentle, non-confrontational approach, which did not assume pathology (Starnino, 2014; Mental health Foundation, 2002). Whilst most of the studies reporting on the phenomena focussed on the interplay between spirituality and symptoms, for some participants, this distinction was not necessarily clear cut and they questioned the ways in which certain experiences were labelled in the first place:

‘What I had to sort out was a religious existential problem and to them it was classic schizophrenia’ (Mental health foundation, 2002: 49).

One of the key factors influencing how people experienced and dealt with the interaction between spirituality and mental health symptoms was to do with how people coped with them and the strategies, frameworks and practices they utilised for this.

## 6. Coping

Coping was an important and prominent theme, which, along with its sub-themes, was highlighted in 34 studies. This theme inter-connects with the others, particularly meaning-making, as this is an important aspect of coping. Because this is quite a broad topic and was often central to people’s experiences of spirituality and mental health it has four sub-themes: Spiritual practices, Spiritual relationship, Spiritual struggles and Preventing suicide.

The theme of coping refers to the many ways in which people utilised their spirituality to help them to deal with the challenges of their mental health problems. Most often this was achieved by utilising various spiritual practices or through cultivation of people’s relationship with their spirituality, faith, God or their spiritual communities. Although often helpful, the process of acquiring the ability to cope was not necessarily easy and could be impeded by symptoms or struggles. However, one of the most striking sub-themes found across a sizeable number of studies, was that for some participants, their faith not only helped them to cope but, without it, they would not have survived.

There were a large variety of ways in which people described how their religion and spirituality helped them to cope with the challenges which arose from their mental health difficulties. These included turning to their own internal world to experience consolation (Koslander & Arvidson, 2006), engaging in positive decision making and taking action to support recovery (Starnino & Canda, 2014), using spiritual beliefs to reduce or cease symptomatology (Buser et al., 2014) and turning to religious and spiritual teachings to help make sense of their problems (Eltaiba & Harries (2015). These approaches could lead to a sense of peace and calm, comfort, support, guidance, strength, self-reliance, forgiveness, hope, purpose and positive self-image (Wilding et al., 2005; Salimena et al., 2016; Heffernan, 2016; Moller, 1999).

The process of coping was often experienced as an active process involving making persistent efforts to engage with spiritual and recovery processes and to gain skills and acquire information. The most important ways which helped people to cope spiritually, were engaging in spiritual practices and their relationship with God or their spirituality.

### 6.1 Spiritual practices

Sixteen studies highlighted the importance of spiritual practices in helping participants to cope with mental health problems. People engaged with a variety of practices, with prayer reported as having particular significance and being a key source of coping, providing strength, maintaining connection with God, promoting gratitude, reducing fear and sustaining a sense of respect and hope (Al-Solaim & Loewenthal, 2011; Eltaiba & Harries, 2015; Murphy, 2000; Corry et al., 2015; Smith & Suto, 2012; Sreevani & Reddemma, 2012; Sullivan; 1993; Mahintorabi et al., 2017). Prayer was also often seen as a way of communicating with God as a ‘direct line to God’ (Mental Health Foundation, 2002: 32). In a study exploring religion and spirituality among young adults with severe mental illness, Oxhandler et al. (2018) found that prayer was one of the most common religious coping strategies amongst participants, as it provided a strategy for resilience and helped them cope with extremely difficult life situations.

Attending church, temples or places of worship or accessing sacred quiet spaces were also important ways in which people could express their spirituality or gain a sense of peace or comfort (Salimena et al., 2016; Macmin & Foskett, 2004; Mental Health Foundation, 2002). Reading religious and spiritual texts was seen as an important way for participants to cope and seek answers: ‘…you could find a solution in the Bible’ (Drinnan & Lavendar, 2006: 324). Meditation and mindfulness were other practices participants said they found helpful to obtain a sense of connection, peace or guidance or to manage difficult experiences (Corry et al., 2015; Nixon et al., 2010). A range of other practices were mentioned across the studies including rituals, ceremony, attending confession, singing hymns or spiritual songs, yoga, meeting religious people and attending formal religious services (Mental Health Foundation, 2002; Sullivan, 1993; Sreevani & Reddemma, 2012; Nixon, et al., 2010; Hustoft et al., 2013).

One of the key benefits of engaging in spiritual practices was that they could strengthen people’s connection with their spiritual lives or their relationship with God.

### 6.2 Spiritual relationship

People’s relationship with God, a spiritual figure or a higher spiritual power was very significant for participants, being mentioned in 21 studies. Many people described this relationship as central to their faith or the most important relationship of their lives and for this reason it had crucial importance for coping during times of illness (Lilja, et al. 2016; Hanevik et al. 2017). This relationship often provided people with a sense that ‘God was still there’ (Lilja, et al. 2016: 4) and cared throughout someone’s times of illness and could be called upon whenever needed (Wilding et al., 2006):

‘I know I matter to God, no matter what, no matter how depressed I am, no matter how much I fail, whatever mistakes I make’ (Russinova & Cash, 2007: 278).

This relationship also provided a great sense of comfort, reassurance, protection, guidance and security, sometimes in almost a parental way (Rieben et al., 2013; Lilja et al., 2016; Drinnan & Lavender, 2006; Hanevik et al., 2017). Participants described how this relationship brought them feelings of peace, strength, courage and the ability to be more positive about themselves (Wilding et al., 2006; Lilja et al., 2016; Murphy, 2000:182; Salimena et al., 2016). God was seen as a salvation or a ‘very best friend’ who helped the participants to cope with challenges and provided understanding, forgiveness and solutions to problems (Muir-Cochrane & May, 2006: 147; Mental Health Foundation, 2002; Corry et al., 2015; Sreevani & Reddemma, 2012; Salimena et al., 2016):

‘It’s God who helps me, I ask him for solutions and slowly he helps me, giving me the answers’ (Salimena et al., 2016: 4).

‘I really don’t look to people. I look to God … God has seen me through everything’ (Sullivan, 1993: 130).

Sometimes people perceived God or a higher power to act directly as a ‘divine intervention’ or ‘divine action’ to assist with recovery, coping or reducing symptoms (Buser et al., 2014: 104; Rieben et al., 2013; Heffernan et al., 2016).

‘…get connected with God, because he’s the only person that can really calm me down and keep me on a level that I need to be’ (Oxhandler et al., 2018).

Spiritual connection was a key theme in Rajakumar et al.’s study (2008) and was expressed through relationships with not only a spiritual figure but also with self, others and nature. They found that these connections helped participants in their study with depression derive a sense of meaning and purpose from their struggles. Although connecting with faith and spiritual communities was mentioned as important in several studies (Mental Health Foundation, 2002; Heffernan et al., 2016; Sullivan 1993) perhaps because of some of the difficulties this could also bring through potential rejection and stigma (Mental Health Foundation, 2002; Baker, 2010), greater prominence was given to a direct relationship with a spiritual entity.

A key finding in Heffernan et al.’s (2016) study was that the role of a genuine reciprocal relationship with a deity was so essential that it influenced many other aspects of people’s experiences. Losing the opportunity to connect with God, spirituality or engage in religious observance in hospital settings for fear of social disapproval impeded this relationship and people’s opportunities for recovery.

Sometimes this spiritual relationship could also bring struggles when God was experienced to be judging or when people felt ashamed of their failures (Lilja et al., 2016). These struggles are described further in the following sub-theme.

### 6.3 Spiritual struggles

The previous themes have already described some of the challenges people experienced during their illness, particularly in relation to service provision, wanting to talk about and make sense of their spirituality, and when people’s spirituality interacted with their mental health symptoms. This theme describes spiritual struggles more generally in relation to coping and highlights that people’s spirituality, practices and relationship with God or a higher power, although often vital and central to their recovery, was not without challenge.

One of the most prominent areas of spiritual struggle was feelings of guilt. Often this was because of being unable to maintain rituals or spiritual practices (Heffernan et al., 2016) and sometimes this was a general existential feeling coupled with shame, feelings of unworthiness or of being a sinner (Lilja et al., 2016; Buser et al., 2014; Rieben et al., 2013; Drinnan & Lavender, 2006; Moller, 1999; Marsden et al., 2007). Other problems related to people’s relationships with their spiritual communities, sometimes feeling excluded or abandoned by them, or to do with the judgements or stigma they might experience due to their mental health problems (Eltaiba & Harries, 2015; Moller, 1999).

Other spiritual struggles included feeling punished by a higher being, loss of personal agency and hope, isolation, negative emotions, negative religious coping and thinking and feeling misunderstood (Yang et al., 2011; Starnino & Canda, 2014; Hustoft et al., 2013; Mental Health Foundation, 2002; Hanevik et al., 2017). Sometimes these struggles led to exacerbation of symptoms (Mahintorabi et al., 2017) or people wanting to give up their faith altogether:

‘I have actually said to God, why don’t you just leave me … It was more comfortable when I didn’t know’ (Mental Health Foundation: 2002: 16).

Wanting to give up completely however, is the topic of the next sub-theme.

### 6.4 Preventing suicide

An alternative name for this sub-theme framed in participants’ own words could be ‘Without my faith I wouldn’t have survived*’* (Mental Health Foundation, 2002: 14; Nixon et al., 2010: 538). This reflects the poignant ways participants expressed the crux of their relationship with their faith or spirituality when dealing with mental health difficulties. Minor variations of this phrase or notion occurred in a surprisingly large number of studies (n=13).

The essence of this theme concerns how people’s faith, spirituality or relationship with God was often the very thing which kept them alive during times of their most difficult struggles with mental health problems. It did this through the prevention of suicide (Hustoft et al., 2013; Al-Sohaim & Loewenthal, 2011; Murphy, 2000; Drinnan & Lavender, 2006; Lilja et al., 2016; Corry et al., 2015; Wilding et al., 2005; Mental Health Foundation, 2002; Oxhandler et al., 2018).

Within this sub-theme, the question of whether to remain alive or not was answered by participants’ faith or spirituality:

‘God has saved my life.’ ‘Probably the biggest impact about my belief in God is when I have been suicidal… my faith has probably been the thing that’s most kept me from hurting myself’ (Rajakumar et al., 2008: 96).

‘If I had no faith, I don’t know how I’d get through it. No faith, no hope, no light at the end of the tunnel. I would end it’ (Drinnan & Lavender, 2006: 324).

Some people reported experiencing a form of divine intervention at the point at which they were about to take their lives, seeing this as a ‘miracle’ or as a sign of God’s care and assurance that their lives remained worthy (Lilja et al., 2016; Corry et al., 2015; Mental Health Foundation, 2002; Wilding et al., 2005). Sometimes it was these experiences which precipitated their recovery and became turning points in their lives:

‘I stood on the edge and ready to jump in and a voice … I felt it was God speaking to me …and I suddenly realised what it was and what I was going to do and then I realised I didn’t want to do that and I think from that point I started to recover’ (Mental Health Foundation, 2002: 13).
