# Supplementary material for: The experiences of spirituality among adults with mental health difficulties: a qualitative systematic review
Source: Epidemiol Psychiatr Sci. 2019 May 3;29:e34. doi: 10.1017/S2045796019000234 (PMC8061134; doi:10.1017/S2045796019000234)
Supplement: Supplementary file 1 [file epssup.zip › S2045796019000234sup001.docx]

**Online Supplement 1: Systematic review exclusion criteria**

| **Exclusion Criteria** | **Further Details and Examples** |
| --- | --- |
| Under 18 years | Children, teenagers, adolescents. |
| Organic disorders | Dementia, Alzheimer’s. |
| Learning disabilities | e.g. Autism spectrum disorders. |
| Drug and Alcohol | Where this is primary focus, including recreational drug use and psychedelic/entheogenic substances. |
| Physical Illness | e.g. cancer, HIV, Aids, cardiac health. |
| Forensic | Crime, homicide, offenders, violence, psychopathy, sociopathy, terrorism. |
| War related | Veterans, soldiers, military, war related trauma, prisoners of war. |
| Natural and Geographic | Natural disasters, geography/earth related events, general disasters, catastrophes, holocaust, slavery, migration, immigration, refugees. |
| Death-related | Death-related anxiety, palliative care, grief, bereavement, suicide, suicidal ideation, self-harm. |
| Birth-related | Pregnancy, motherhood, post- and peri-natal depression. |
| Sexuality-related | Abuse, rape, transgender, gay, lesbian, homosexuality. |
| Relationships | Relationship problems, couples counselling, domestic violence. |
| Specific religious/ spiritual phenomena | Religious conversion, possession, exorcism, paranormal themes, ghosts, spirits, psychic phenomena. |
| Staff or carer perspectives | Expert/clinician/clergy perspectives, where primary focus is not perspectives of people with mental health difficulties. |
| Work/Employment | Including staff and professional perspectives. |
| Intervention | Therapy, group. |
| Information Sources | Opinion pieces, autobiographies, theoretical studies, dissertations, theses, books, book chapters, book reviews, quantitative studies (including questionnaire/scale/ assessment studies), case studies involving three or less participants, studies comprising data prior to 1980. |
